# Upcoming progress of transcriptomics studies on plants: An overview

**DOI:** 10.3389/fpls.2022.1030890

**Published:** 2022-12-15

**Authors:** Parul Tyagi, Deeksha Singh, Shivangi Mathur, Ayushi Singh, Rajiv Ranjan

**Affiliations:** Plant Molecular Biology Lab, Department of Botany, Faculty of Science, Dayalbagh Educational Institute, Agra, India

**Keywords:** NGS, transcriptome analysis, gene function, molecular markers, secondary metabolites

## Abstract

Transcriptome sequencing or RNA-Sequencing is a high-resolution, sensitive and high-throughput next-generation sequencing (NGS) approach used to study non-model plants and other organisms. In other words, it is an assembly of RNA transcripts from individual or whole samples of functional and developmental stages. RNA-*Seq* is a significant technique for identifying gene predictions and mining functional analysis that improves gene ontology understanding mechanisms of biological processes, molecular functions, and cellular components, but there is limited information available on this topic. Transcriptomics research on different types of plants can assist researchers to understand functional genes in better ways and regulatory processes to improve breeding selection and cultivation practices. In recent years, several advancements in RNA-*Seq* technology have been made for the characterization of the transcriptomes of distinct cell types in biological tissues in an efficient manner. RNA-Seq technologies are briefly introduced and examined in terms of their scientific applications. In a nutshell, it introduces all transcriptome sequencing and analysis techniques, as well as their applications in plant biology research. This review will focus on numerous existing and forthcoming strategies for improving transcriptome sequencing technologies for functional gene mining in various plants using RNA- Seq technology, based on the principles, development, and applications.

## Introduction

Since the advent of the post-genomic era, numerous omics approaches have been developed, such as genomics, transcriptomics, metabolomics, and proteomics. As one of these technologies, transcriptomics is the second earliest and most commonly used (Lockhart and Winzeler, 2000; Levy and Myers, 2016). Transcriptomics studies focus on the transcriptome. Because of its high throughput, improved precision, and cost effectiveness during the past two decades, genomic sequence databases have increased massively (Lathe et al., 2008; Jain, 2012; Karsch-Mizrachi et al., 2018). However, the complex mapping of a genome to various phenotypes, tissues, developmental stages, and environmental factors continues to pose a major challenge in molecular biology. A more in-depth understanding of gene regulation transcripts and expression is not only difficult but is also at the core of the problem. Transcriptomics has been extensively studied in a variety of organisms and provides critical insights into gene structure, expression, and regulation (Lowe et al., 2017). In recent years, transcriptomics research has grown tremendously due to rapid progress in sequencing technologies (Wang et al., 2016; Abdel-Ghany et al., 2016).

In plant research, there are some significant differences between transcriptomics and genomics. First, genome assembly is more complex and costly in plant research compared to transcriptomics (RNA-*Seq*); in the absence of a reference genome, the transcriptome can be used to assess an organism or plant’s overall transcriptional activity. Second, the transcriptome changes over time and space because it includes information on secondary metabolic pathways as well as variations in gene expression at different times and spatial regions. According to research, the accumulation of biologically active compounds in different plant tissues depends on the gene expression levels of that tissue as well as the time they are produced. This is because plants have various growth conditions and periods, even within the same species. As a result, the transcriptome outperforms the genome in terms of identifying genes related to therapeutic plant components (Wang et al., 2009). These differences are important for studies on plant functional genome mining, gene regulatory domains development, genetic diversity (dominant and recessive genes), and bioactive compounds (Tyagi and Ranjan, 2021; Tyagi et al., 2022).

Transcriptome study approaches have progressed from basic DNA microarrays platform to RNA-*Seq* technology in recent times due to the continual developments in sequencing technology (Mironova et al., 2015). It has several perks including high sensitivity, high throughput, and efficacy, which may be used to analyses a complete transcriptome without a genomic reference sequence. RNA-*Seq* technology is a popular sequencing approach in molecular biology, biotechnology, and bioinformatics (Sun and Wei, 2018). This method has been widely exploited in model plants, including *Arabidopsis thaliana (* Zhang et al., 2019 *)*, *Oryza sativa* (Li et al., 2012; Pradhan et al., 2019; Yang et al., 2021), *Zea mays* (Xu B et al., 2014; Liu et al., 2019; Xu et al., 2021), *Rehmannia glutinosa* (Ma et al., 2021), *Polygonum cuspidatum* (Wang et al., 2021), *Asarum sieboldii* (Chen et al., 2021), *Calotropis gigantea* (Hoopes et al., 2018). Transcriptome can be defined as the total number of RNA molecules transcribed from a particular tissue or cell at a given functional or developmental stage, which includes mRNA (messenger RNA) and nc-RNA (non-coding RNA). They are referred to as “bridges” because they accurately manage the transport of genetic information from DNA to protein (Costa et al., 2010), whereas non-coding RNA influences gene expression, protein synthesis, and various cellular processes at several levels (Kumar et al., 2016). Understanding transcriptomics thus enhances research with reference to the functions of cells, tissues, and organisms. RNA-Seq is a fairly new technology that measures all the biological amounts of the transcriptome. This makes it easier to study the transcriptome (Wang et al., 2009).

Computing and analysing RNA-seq still remains a challenging task. Accurate alignment of sequencing reads, and accurate expression level inference are some of these challenges. In RNA-seq, the central computational challenge is accurately and efficiently assigning short sequencing reads to their transcripts and determining gene expression based on that information. There have been some benchmark studies conducted, but they have generally been conducted using simulated RNA-seq datasets or RNA-seq datasets focusing only on long RNAs, such as messenger RNAs and long noncoding RNAs. Therefore, they did not assess the suitability of these tools for measuring total RNA in datasets that included small RNAs like transfer RNAs and small nucleolar RNAs. This review paper focuses on various methodologies, including transcriptome sequencing. Additionally, it provides an overview of the advancements made in using the RNA-Seq approach in plant research, including the molecular markers identification, functional gene expression analysis, biosynthetic pathways of secondary metabolite, and mechanisms for plant development.

## Techniques of next generation sequencing (NGS) and their potential applications

### RNA-seq: A history and evolution

Sequencing of events is a crucial comprehension method for a variety of reasons. Researchers of various abilities can efficiently organize material and ideas using sequencing structures. It is crucial to problem-solving in all subject areas, including science and social studies. So, differences of sequencing are coming according to generation, in first-generation sequencing platform, enabled sequencing of clonal DNA populations, in second generation sequencing, massively increased throughput by parallelizing many reactions, and in third generation sequencing, allow direct sequencing of single DNA molecules. In the 1970s, Walter Fiers and colleagues marked the start of RNA-Seq studies by sequencing the MS2 bacteriophage (3,569 nucleotides) whole transcriptome (Crowgey and Mahajan, 2019). RNA is highly unstable and susceptible to breakdown by cell membrane-associated RNases due to its single-stranded structure. Therefore, extensive efforts are required to sequence the whole transcriptome accurately. The discovery of reverse transcriptase (Zhang, 2019) enables the transformation of messenger RNA and non-coding RNA into stabilized DNA. The reverse-synthesized DNA is referred to as complementary DNA (cDNA). Sanger, a British scientist, invented DNA sequencing technology in 1975 (Zhang, 2019; Sanger and Coulson, 1975). Iscove (Hwang et al., 2018) used the technique of RNA-Sequencing PCR, which exponentially amplifies cDNA. As a result of these pioneering efforts, microarray (chip) technology was developed (Bolón-Canedo et al., 2019). Through Arrayed technology, hundreds of known partial DNA sequences can be mounted on nylon membranes or solid support slides using molecular hybridization technology. Numerous genes can be quantitatively detected through hybridization. The reverse synthesis of cDNA can rapidly be used to sequence the transcriptomes of different biological samples, making microarrays an excellent way of exploring gene function (Clark et al., 2002). As of now, the technology is primarily used to study known genes, rather than unknown genes. Additionally, the microchip has difficulty detecting numerous transcripts generated by alternative splicing.

Sanger sequencing is an expensive, laborious, and time-consuming technique. NGS sequencing, however, has met a number of critical needs for molecular biologists since 2006, when it was introduced. In comparison to first-generation sequencing technology, NGS is fast, high-throughput, and economical. It is possible to sequence millions or billions of nucleic acids simultaneously, enabling the analysis of transcriptomes and genomes of all species using NGS. As of today, NGS is used for constructing different organism genomes for the purpose of collecting the complete gene sequences of various species of plants, including SARS-CoV-2 (Li et al., 2020) and *Chosenia arbutifolia* (Khoar), a korean plant (Chen et al., 2014; Mei et al., 2016; Feng et al., 2019). Next generation sequencing is predominantly employed to sequence and analyze messenger RNA (mRNA) and small RNA (small RNA) from the transcriptome. As indicated in **Table 1** (Zhang et al., 2016), the Roche 454, Illumina Solexa, and ABI SOLiD are all prominent next-generation sequencing platforms.

**Table 1:** Comprehensive overview of various next-generation sequencing (NGS) technologies.

Name of Sequencer	Sanger Sequencing	Roche 454 sequencing	Solexa-Illumina	ABI SOLiD	SMRT	Nanopore
**Based on Principle**	“Dideoxy chain termination”	“Sequencing by synthesis”	“Sequencing by synthesis”	“Sequencing by ligation”	“Sequencing by synthesis”	“Electrical signal sequencing”
**Advantage**	Higher sensitivity to detect low-frequency variants	Long read length, ferments are generated in large number	Enables a wide variety of applications, allowing researchers to ask virtually any question related to the genome, transcriptome, or epigenome of any organism	High accuracy, Coverage is more than 30x, Detecting targeted re-sequencing, and transcriptome sequencing	Short time-consuming and no need for PCR amplification	Ability to produce ultra-long reads
**Disadvantage**	Sequencing could only small DNA sequence, higher cost, and minimum high throughput	More than 6 error rates with polybases, high cost, and low throughput	Short reads length, low multiplexing capability, only highly trained person can operate	Short read length, prone to chain decoding error	High cost and minimum high throughput	High single-base error rate and long sequencing time
**Length of base pair**	15-40bp	300-800 bp	2×150bp	~75 bp	8-15 to 40-70kb	500bp-2.3Mb
**Developed by and year**	Frederick Sanger and colleagues in 1977	Shankar Balasubramania and David Klenerman in 2005	Shankar Balasubramanian and David Klenerman in 2006	Robert C. Martin (also known as Uncle Bob) in 2006	Christian Henry, and John F. Milligan in 2004	Hagan Bayley Clive G. Brown et al., in 2005
**Run time**	4-5hrs	24 hrs	6-7 days	6-7 days	~6 hrs	10 minutes
**Output data**	1.9~84 Kb	~7 Gb	600 Gb	120 Gb	5~10Gb	More than 50 Gb
**References**	Sanger et al., 1977	Zhang et al., 2016	Wang et al., 2009	Xu, 2018	Li et al., 2018	Ma et al., 2019

In 2005, Roche introduced the high-throughput Roche 454 sequencing technology, based on pyro-sequencing technique (Margulies et al., 2005). A longer read length and short run time make 454 sequencing stand out from other NGS technologies. This approach is non-fluorescent and does not require nucleic acid probes. However, it has the potential to introduce errors such as deletions or insertions during the process of sequencing (Liang et al., 2017). The method utilized by Solexa (Illumina) primarily rely on the principle of “sequencing by synthesis”, in which DNA fragments are randomly linked on a flow cell. Following extension and amplification, the surface of the glass generates millions of clusters containing thousands of identical DNA fragments. Sequencing of labeled dNTPs (fluorescence) is performed on the stretched DNA strand. This technology permits the use of synthetic probes and reference sequences in genome-wide expression analysis (Liu et al., 2012).

There are a few drawbacks to the platform, including its shorter read length as well as its complexity in assembling reads from scratch. With solid sequencing (2007), magnetic beads are used to sequence data in a highly parallel manner. This approach allows massive DNA amplification without incurring high costs because of constant ligation and development of fluorescently labeled oligonucleotides (Li and Xu, 2019). While high precision is the significant benefit of this technology, the primary disadvantage is that it is prone to chain decoding errors if an error occurs, as mentioned in **Table 1**.

Third-generation sequencing has been enabled ‘by improvements in sequencing technologies’. In addition to amplification requirements for NGS platforms, template relocation and mismatches in nucleotides and GC percentages are common challenges related to these approaches. These challenges reduce the accuracy and completeness of our sequencing data. Because third-generation sequencing offers such long reads, it has been widely used in structural variation, methylation, transcriptome and genomic analysis, among others. SMRT (Single- molecule real-time) and Nanopore sequencing have recently become key technologies for third-generation sequencing (Ma et al., 2019). As a sequencing method, the SMRT employs the “synthesis-based principle.” SMRT technology has two unique properties compared to classical methods. This was followed by adding and linking the fluorescent group to the phosphoric acid (H_3_PO_4_) group to eliminate the background noise. As a second benefit, there is no need for amplification and the SMRT allows for more precise measurements through self-correction (Li et al., 2018). On the other hand, SMRT sequencing technology has several disadvantages, one of which being the integration of random errors. By using circular consensus sequencing (CCS) and improving sequence coverage, it is now possible to increase the accuracy of a single read to 99.8% in comparison to second generation sequencing (Wenger et al., 2019).

Nanopore is a next-generation SMRT sequencing platform that determines the base composition mostly through changes in electrical impulses. Nanopore sequencing is more cost-effective and offers longer reads than other platforms. However, the major drawback of this technology is its high error rate which ranges from 5-20% (Huang et al., 2021). Despite its advantages, it has some disadvantages, including a high probability of random errors and single-base errors as mentioned in **Table 1**. Using the above approaches without a reference sequence can be cost-prohibitive and time-consuming. RNA-Seq is a crucial part of NGS technology and an important tool for transcriptome analysis. It overcomes the limitations of microarray analysis and provides a greater understanding of transcriptome research.

### Sample preparation and assembly for RNA-*seq*

**Figure 1:**
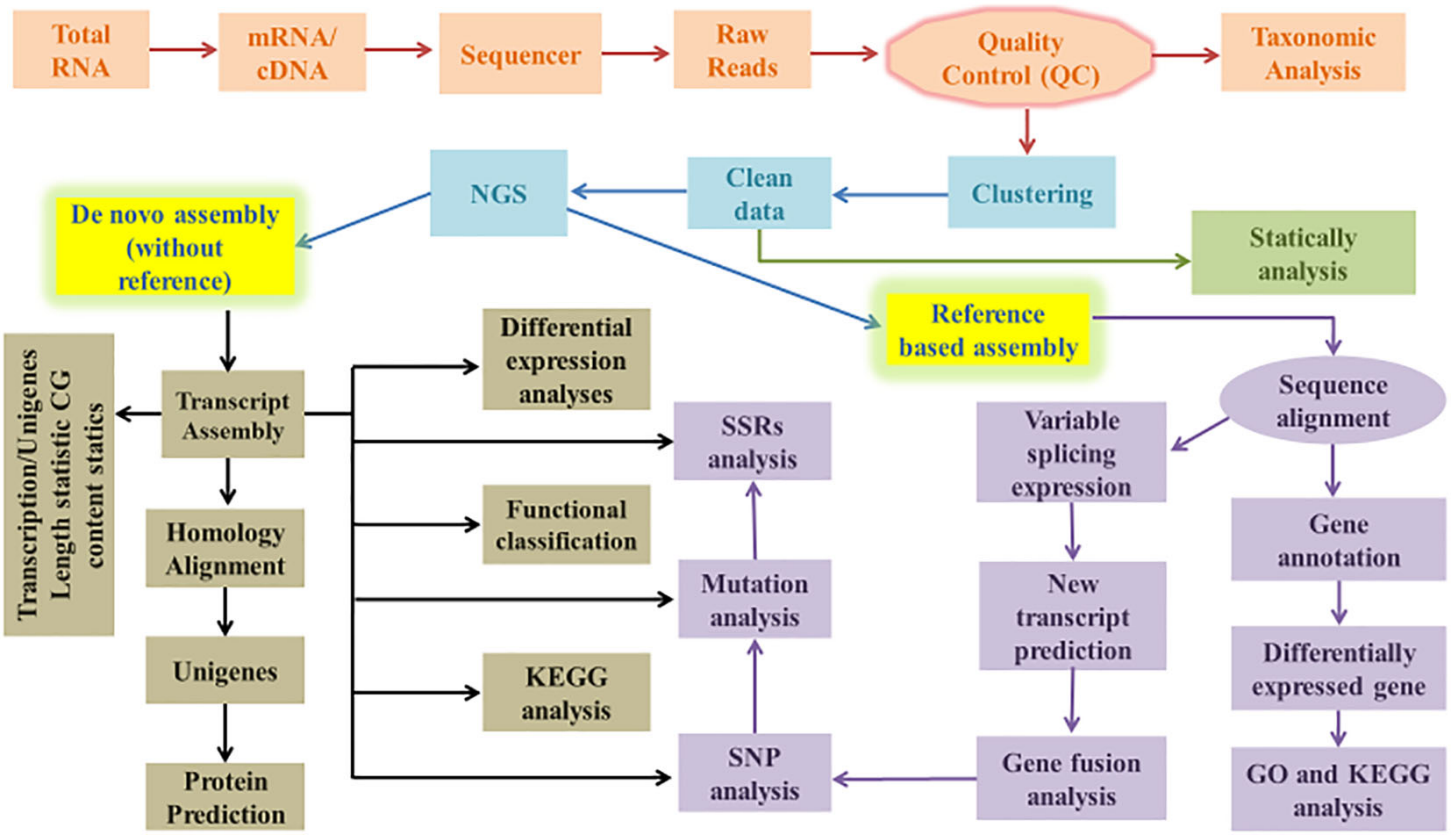
Flow chart of transcriptome (*De novo* and reference based) data analysis.

The total RNA from biological samples is isolated using kits or manually (TRIzol) protocols (Drygin et al., 2021). m-RNA was extracted from total RNA “Experiments should be employed in triplicate form.” The enriched mRNA was fragmented and converted into first-strand cDNA, which was then followed by second-strand generation, A-tailing, adapter ligation, and a limited number of PCR amplifications of the adaptor-ligated libraries. The amplified libraries were analyzed on the Bio-analyzer according to the instructions of the manufacturer. Quantification and qualification of the library were performed using any sensitivity kit by the Nanodrop spectrophotometer. NGS for cDNA of all plants was performed by library on any sequencing platform according to samples. The raw reads were first filtered to exclude the reads containing adaptors or with ambiguous nucleotides (‘N’). Below than 20% Q < 20 nucleotide bases were also trimmed. The obtained high-quality (HQ) clean reads were used to construct a sequence assembly using Trinity (Tyagi and Ranjan, 2021) or any other software. After removing duplicate Trinity-generated sequences with the TGICL, clusters and unigenes were recovered, as illustrated in **Figure 1**. Cogent creates a k-mer profile of non-redundant (NR) transcripts, calculates pairwise distances, and then categorizes transcripts into families based on k-mer similarity. Using any of the graph techniques, each transcript family was further reconstructed into one or more distinct transcript model (s).

Transcriptome assembly is required for a variety of downstream analyses. Raw transcriptome data is error-prone due to the vast amount of transcriptome data (Nagalakshmi et al., 2008). Thus, selecting assemblies based on distinct transcriptome data and research ideas is important. The primary thing to consider when assembling is whether there is a reference sequence available; assembly can be classified into two types, reference-based assembly and *de novo* assembly as shown in **Figure 1**. It is impossible to carry out the reference assembly for most plants because their genomic sequence data is frequently lacking; therefore, many non-model species are not provided with genomic sequence information. The only assembly approach appropriate for non-model plants is *de novo* assembly, which is depicted in **Figure 1**.

### The assembly and function annotation process

Software such as Cufflinks and Scripture (Guo et al., 2021) is used to assemble genomes, whereas Oases, Trinity, Rnnotator (Martin et al., 2010), and SOAPdenovo-Trans (Madritsch et al., 2021), are used to assemble *de novo* genomes (Martin et al., 2010; Schulz et al., 2012; Grabherr et al., 2013; Trapnell et al., 2013; Xie et al., 2014). Assembling a single K-mer method with SOAPdenovo-Trans and Trinity is one stage of the *de novo* (without reference) assembly, while multiple K-mer methods are assembled with Oases and Rnnotator. Numerous k-mer methods can be utilized for obtaining huge transcripts of data, but when the findings of numerous k-mer methods are merged, a huge amount of duplicated data is generated, thus increasing the error rate and data complexity. As a result, at present time, a single K-mer assembly method is the most significant for improved precision that can be adopted, as mentioned in **Table 2** (Strickler et al., 2012). SOAPdenovo-Trans connect to the contigs using an unknown nucleotide base (N). This approach is less accurate than the other three software programs in terms of assembly correctness, does not produce longer transcripts, and has a lower average accuracy rate (Lu, 2013). In recent years, Trinity has become an increasingly popular *de novo* assembly program due to its ability to produce accurate and efficient assembly outputs. In that respect, Scripture and Cufflinks use sensitive and conservative assembly methods, respectively. Despite the superior quality of Cufflinks’ assembly, Scripture’s transcript quantity greatly exceeds that of Cufflinks, as shown in **Table 2**. Cufflinks can provide higher accuracy and quality assembly results by using high-quality reference sequences.

**Table 2:** List of softwares used in *De novo* assembly pipeline with their functions.

Name of Software	Raw data	Data filtration	Output
**Through Sequencer (** Leng et al., 2012 **)**	RNA-*Seq* Raw Reads	**Short Reads-** Obtained from the common NGS platforms, including Illumina, SOLiD and 454, are often very short bases (35-500 bp). **Long Reads-** Oxford Nanopore/PacBio Sequencers can sequence up to long 5 to 100Kb reads.	Massively parallel millions to billions sequence that offers high- throughput, scalability, and takes lesser time.
**FastQc (** Andrews, 2010 **)**	Quality Check (QC)	**Quality assessment-**Evaluate the raw read quality, identify the adaptor contaminations, and identify low quality samples	**Good/Bed-** According to Phred Score quality (Q-value)
**Trimgalore**	Read clean up (If contaminated)	**Trimming-** Removes the bad bases (adaptor sequences and low-quality bases) at start and end of the reads **Filtering-** Removes contaminants, low complexity reads (repeats), short reads less than 20 bases	**K-mer-** Shorter nucleotides than the read length ***De Bruijn* graph-** Several transcriptome assembly programs. Every path in the graph denotes a potential transcript for transcriptome assembly.
***De novo* assembly (** Zhao et al., 2011 **)**	Trinity (Henschel et al., 2012)	**Quality/Phred Score quality (Q-score)-** prediction of the probability (P) of an error in base calling. Q(phred) = -10 log_10_P Or P = 10^–Q/10^	***De novo* Assembler-** A novel method for the efficient and robust *de novo* reconstruction of transcriptomes. **Software modules-** Inchworm, Chrysalis, and Butterfly
**RSEM (** Li and Dewey, 2011 **)**	Transcript Abundance Estimation	Assembly Statistics	**N50** length is defined as the shortest sequence length at 50% of the transcriptome
**Cd-hit (** Suzek et al., 2007 **)**	Transcript clustering	Generate unigenes	Group of transcript sequences
**edgeR (** Robinson et al., 2010 **)**	Differential Expression Analysis	*edgeR* can be applied to differential expression at the gene, exon, transcript, or tag level. In fact, any genetic feature can be utilized to calculate read counts. There are two testing methods: likelihood ratio tests and quasi-likelihood F-tests.	The key abilities of package, and then gives several fully worked case studies, from counts to list of genes
**TransDecoder (** Wang et al., 2009 **)**	Coding DNA Prediction	CDS prediction from unigenes	Segments of a gene’s (mRNA) that code for protein.
**Blast2GO (** Martin et al., 2004 **)**	Gene Ontology (GO)	Mapping and annotation	In detail, describe a gene/gene product, including three main characteristics: molecular function (MF), Biological process (BP), cellular compound (CC)
**Trinolate (** Wang et al., 2009 **)**	Functional Annotation	(COG) Clusters of Orthologous Groups (for prediction of individual proteins function), Ven diagram (to identify common genes of all software), Pfam domain (to identification of protein family), Volcano plot (gene expression), Scattered plot (for normalization of obtained values), Heatmap (for highly significant differential expressed genes)	Portion identification and Gene prediction (process of collecting information about and describing a gene’s)
**KAAS (** Moriya et al., 2007 **)**	Pathway Prediction	Pathway analysis against KEGG databases	Identification of biological functions
**DESeq2 (** Love et al., 2017 **)**	Differential Expression Analysis	Normalization, differential analysis and visualization of high- dimensional count data	Count matrices can be collapsed using collapse Replicates, which helps to combine counts from technical replications into single columns.

Gene function annotation is the process of identifying the functions related with individual gene based on existing data by comparing unidentified gene sequences to those published in public databases. GenOntology (GO) (Yu, 2020) and KEGG (Kyoto Encyclopedia of Genes and Genomes) (Yousef et al., 2021) are the two widely used approaches for functionally categorizing gene functions A GO is divided into three categories, namely biological processes (BP), cellular compounds (CC) and molecular functions (MF). Unigene sequences are commonly annotated by databases such as Non-Redundant (NR), Protein Sequence Database (Zhao et al., 2021), GO, KEGG, Clusters of Orthologous Groups (COG) (Lee and Lee, 2021), Pfam (Mistry et al., 2021), NCBI Nucleotide Sequence Database (Schoch et al., 2020), and Swiss-Prot database (Liu FX et al., 2018).

## Application of transcriptome sequencing

The RNA-Seq method provides insight into gene expression under a variety of environments and enables the discovery of new genes (Clamp et al., 2007; Mortazavi et al., 2008; Shendure, 2008; Pickrell et al., 2010; Touch et al., 2010; Filichkin et al., 2010), which in turn help us understand how cells function and how they function metabolically. The primary benefit of RNA-Seq is that it allows the comparison of gene expression patterns across samples.Before the advent of deep sequencing technology, the main means of measuring the expression levels of different genes was the microarray (Barrett et al., 2013; Anita and Caterina, 2018). However, hybridization techniques have low sensitivity, making it difficult to identify low-abundance targets and are incapable of finding tiny changes in the expression level of the target gene (Birney et al., 2007). Therefore, RNA-*Seq* is more accurate than microarray. RNA-*Seq*, in general, may measure the absolute number of each molecule in a cell population and directly compare the results between tests. Second, RNA-*Seq* promotes new gene discovery. The annotations to transcripts in existing databases may not be comprehensive. The RNA-*Seq* results might self-assemble without the need for known genome annotations, facilitating the discovery of new genes (Zhao et al., 2014).

Third, RNA-Seq has shown satisfactory results in detecting sequence differences, including the identification of fusion candidate genes and the investigation of coding sequence polymorphisms. Because of alternative splicing, a single gene can produce many mRNA transcripts, each of which can be translated into a distinct protein with a different function. Alternative splicing is universal, in eukaryotic species. The introduction or removal of various introns and exon regions during the splicing process forms different mRNA precursor obtained during gene transcription. Including the sequences spanning the splice junction region, sequences of all transcripts could be found with appropriate sequencing depth in RNA-*Seq*. The depth is defined as the ratio of the total number of bases (bp) retrieved by sequencing to the genome size, and it is one of the indicators used to evaluate the quantity of sequencing. Lastly, the main aspect of transcriptomics study is the finding and analysis of non-coding RNA (nc-RNA), as shown in **Figure 1**.

### Single cell RNA sequencing technology (scRNA-*seq*)

In recent years, single-cell RNA sequencing (scRNA-seq) technologies have revolutionized the way we think about biological systems, as they are able to provide a high degree of spatial and temporal resolution of analyses (Zhang et al., 2019). Through scRNA-seq of plants, it is possible to identify new cell types and reveal how the different cell types interact spatially and developmental revealing both common and rare cell types and cell states. By using single-cell RNA sequencing technology (scRNA-seq), stem cells can be systematically studied at a cellular and molecular level to gain insight into their differentiation trajectory (Islam et al., 2014; Macosko et al., 2015). An early developmental stage transcriptome was described in 2009 based on a next-generation sequencing platform as the first example of single-cell transcriptome analysis (Tang et al., 2009). High-resolution global views of single-cell heterogeneity have become increasingly popular since this study. A critical aspect of this study is analyzing the gene expression differences (Hwang et al., 2018). When analysis is conducted on individual cells, it may be possible to detect rare populations that may not be detectable in pooled analysis. Furthermore, recent advances in immunological research techniques and bioinformatics pipelines have made it possible for researchers to deconvolute highly diverse immune cell populations (Shalek et al., 2013). Additionally, scRNA-seq is increasingly used to analyze myoblast differentiation (Trapnell et al., 2014), lymphocyte fate (Stubbington et al., 2016), and early development (Petropoulos et al., 2016).

Advances in scRNA-seq technologies have opened new possibilities for uncovering the cellular and molecular differentiation trajectory of plant stem cells. The first application of single-cell technologies is to identify cell subtypes in heterogeneous populations of cells. To date, the Arabidopsis primary root tip is the most studied tissue using single-cell RNA sequencing. By analysing briTRIPLE mutants at single-cell resolution, Graeff et al., 2021 studied the impact of brassinosteroids (BR) signalling in Arabidopsis root tissue. In this study, the researchers found that BR signalling does not affect cell proliferation or cellular development, rather it promotes cellular anisotropy and cell division plane orientation. It is possible to identify phenotypic variations among cell types through single-cell sequencing profiling. ScRNA-seq was used to examine the phenotypes of epidermal cells in the mutant, including root hair deficient cells (rhd6) and glabrous2 cells (gl2). Cell identity phenotypes were identified based on generated data. A study of rice radicals using single-cell sequencing and chromatin accessibility was conducted by Zhang et al., 2021. The development trajectories of epidermal cells and ground tissues were reconstructed using root tip cell profiling, which provided insight into the mechanism that controls cell fate determination in these lineages. Transcriptome profiles and marker genes for these cell types were uncovered through further analysis.

Furthermore, scRNA-seq has been applied to crop improvement in maize by highlighting transcriptional differentiation at high resolution in maize cells. An analysis of 12,525 maize ear cells using scRNA-seq technology was recently published by Xu et al. (2021). A scRNA-seq map of an inflorescence was generated as a result of this profiling. By identifying genetic redundancy in maize, establishing gene regulatory networks at the cell level, and identifying key loci with high ear-yielding characteristics, the generated data can help promote maize genetics. A similar study was conducted by Bezrutczyk and colleagues in the same year, using scRNA-seq to study bundle sheath (BS) differentiation in maize. In maize, single-cell sequencing profiling helped identify cells with unique characteristics on the adaxial side, enhancing the possibility of bioengineering the plant (Bezrutczyk et al., 2021). As single-cell transcriptomics is increasingly applied to several plant species, future research will indeed introduce it to crop species. This will pave the way for its incorporation into applied plant research, which could benefit our agricultural systems in the future.

### Functional gene extraction from plants

To withstand external pressures and adapt to their environments, medicinal plants have evolved a plethora of regulatory mechanisms. A functional gene mining technique identifies key enzymes, pathways, and regulatory mechanisms in plants, thus helping us better understand their molecular biology. Xu WR et al. (2014) analyzed *Vitis amurensis* (Amur grape) transcriptome using the Illumina GA-II sequencing platform and found that in cold regulation a total of 6,850 transcripts involved. There were 3,676 as well as 3,147 copies of transcripts that were upregulated and downregulated, respectively, and 38 key TF families that were implicated in cold regulation. The results of this study provide a foundation for further research into the mechanisms involved in Vitis species’ cold stress tolerance. Pragati et al., 2018 reported that a total of 221,792 and 161,733 transcripts in which 141,310 and 113,062 unigenes were obtained from leaf and root tissues of *Aloe vera* (Gwarpatha), respectively, were used on the Illumina platform. It has been determined that 16 genes are involved in the production of lignin, carotenoids saponins and anthraquinone. The results of this study will be useful in future research into genes involved in secondary metabolite biosynthesis and metabolic regulation in *A. vera* and other Aloe species.

The transcriptome of *Paeonia suffruticosa* was sequenced and examined in 2012 by Mutasa-Gottgens and colleagues, and 81,725 copies of unigenes associated with drought resistance were found. It has been predicted that genes associated with hormone signaling pathways are important for drought adaptation and setting framework to study *P. suffruticosa’s* drought stress response mechanism in the future. Singh et al. (2017) sequenced the *Trillium govanianum* transcriptome using the Illumina sequencing platform, collecting 69,174 transcripts, and discovering many genes involved in steroidal saponin production and biosynthetic pathways of various secondary metabolites. Researchers identified tissues (leaf and fruit) as the primary sites for producing steroidal saponins in the biosynthesis of terpenoids, brassinosteroids, carotene, flavonoids, steroids and phenylpropane. Genetic manipulation is valuable for the identification of biologically active metabolites, as well as for the development of molecular markers that are functionally related to the identified metabolites. A transcriptome sequence of *Polygonum minus* was published by Loke et al. (2017), which revealed 188,735 transcripts. They also reported 163,200 (86.5%) *P. minus* transcript similarity matches, the vast majority of which were with *Arabidopsis* transcripts (58.9%). Root and leaf tissues have improved metabolite pathways. The findings will contribute to the development of this species’ genetic resources.

*Callerya speciosa* genome sequencing was conducted by Li et al. (2016) using Illumina’s platform through which 161,926 unigenes and 4,538 differentially expressed genes were obtained. Store roots may be implicated in starch synthesis, cell wall loosening and light signaling. Additionally, they may play a role in the development of store roots. Using these findings, subsequent research was conducted on growing *C. speciosa* roots, producing therapeutic substances, and breeding the plant. In a study by Hou et al. (2018), 56,392 unigenes and 4,585 significant DEGs were found in *Cornus officinalis* leaf and fruit tissues using next-generation sequencing (NGS). A total of 1,392 genes were up-regulated in fruit tissues, while 3,193 genes were down-regulated. Most DEGs are involved in the regulation of secondary metabolism and the production of terpenoids. This knowledge contributes to the understanding of plant metabolism and gene expression. The phenolic compound rosemarinic acid has antimicrobial and antioxidant properties and is physiologically active. It was reported that *Dracocephalum tanguticum* revealed 151,463 unigenes in its transcriptome by Li et al. (2017). A total of 22 genes are predicted to be involved in the biosynthesis of rosmarinic acid, providing references for future research on rosmarinic acid biosynthesis genes.

### Development of plant molecular markers

SSR markers are a frequently deployed form of microsatellite DNA marker. A tandem repetition sequence consists of 1-6 nucleotide base pairs, with the most common sequence being di-nucleotide repeats. These markers were polymorphic and different numbers of tandem repetitions were associated with them. Historically, SSRs have been used in creating genetic maps, defining genetic diversity, mapping genes, and identifying parental ties due to their high polymorphism, simplicity, codominance, and easy detection. To understand the genetic diversity within asparagus species, Kapoor et al. (2020) used SSR markers to study asparagus varieties grown in different regions of northwest India. There were more than 120 alleles amplified, ranging between 3 and 8, with an average number of five alleles per marker. The lengths of the alleles ranged from 90 to 680 bp. Based on genetic diversity analysis, most Asparagus varieties have a conservative genetic base, except for *A. adscendens*, which indicates that this species has an extensive genetic base.

Future hybridization and conservation of Asparagus species are likely to be affected by these findings. Bhandari et al. (2020) used Illumina paired-end sequencing technology to create new SSR markers for *Salvadora oleoides* (Bada Peelu). From 21,055 microsatellite repeats, they developed 14,552 SSR markers, and randomly selected and confirmed 7,101 SSRs; 94 primers exhibited polymorphisms, and 34 primers failed to amplify. This study provides a foundation for future research on *S. oleoides*.

A HiSeq 4000 sequencing platform was used to analyze transcripts from *Populus alba* (root, leaf, and stem) (Dinh et al., 2020). A total of 11,343 EST-SSRs were identified, of which 101 primer pairs (forwards and reverses) were selected for polymorphism validation. Polymorphisms in populations were discovered by amplifying DNA fragments with 20 primers. Conservation, restoration, and management strategies can benefit greatly from this conclusion. Wang et al. (2020) sequenced and analyzed the transcriptome of *Gastrodia elata* and identified 34,322 unigenes. In 2,007 unigenes (5.85%), at least one SSR was present.

There were 498 detections (21.67%) of a repeating pattern of AG/CT among these SSRs. As a result of this research, Lade et al. (2020) have gained deeper insight into the molecular mechanisms that regulate the growth, development and metabolism of *G. elata* (Tianma). Total 96 sample of *Tinospora cordifolia* were gathered from 10 different geographically diverse locales of the India. In *T. cordifolia*, a total of 268,149 transcripts were assembled. Amongst them, 7,611 SSRs were identified. Tc16, Tc17, Tc31, Tc38, Tc59, Tc60, Tc129, Tc106, Tc130, and Tc131 were shown to contain genetic diversity potential. The potential markers SSR-18, TcSSR-37, TcSSR-59, TcSSR-92, TcSSR-123, and TcSSR-126 have been identified. Genetic enhancement of *T. cordifolia* will be assisted by these components and the newly discovered SSR markers. A comprehensive genetic analysis of two *Menispermum* species was conducted by Hina et al. (2020) using Illumina’s transcriptomic platform and *de novo* assembly. Sum of 521 polymorphic EST-SSRs were found out of a total of 53,712 and 78,921 unigenes. The newly designed EST-SSR marker was also shown to be highly transferrable throughout the *Menispermum* species studied. In order to genetically map *Menispermum* populations, these unique microsatellites will be used. A total of 86,195 unigenes were identified in *P. lactiflora* by using microsatellite software, while 21,998 SSR sites were identified dispersed over 17,567 unigenes. Among the 100 primer pairs, 45 were selected at random and amplified bands of polymorphism. For the cluster analysis of sixteen *P. lactiflora* variations, these 45 primer pairs were used. Molecular markers-assisted breeding with *P. lactiflora* will be facilitated by the novel SSR marker.

### The metabolic pathways of secondary metabolites

Secondary metabolites are often the most significant components of plants. They play an essential part in the process by which plants adapt to their surroundings and build up a defense mechanism against the impact of various stress. There are many factors that affect the accumulation of secondary metabolites, including the growing environment as well as the developmental stage of the part from which it produced. In various stages of development, transcriptomics is used to investigate pathways of biosynthesis, which lead to secondary metabolites, and to mine genes involved in biogenesis. A scientific foundation has been laid for determining how plants accumulate and utilize active components. *Entada phaseoloides* has been used for medical purposes for centuries. Traditional medicine makes extensive use of the stems due to the wind-dampness-eliminating and anti-inflammatory properties that these stems possess. The triterpenoid saponins found in *E. phaseoloides* are the compounds with the highest level of physiological activity. *E. phaseoloides* root, stem, and leaf tissues to uncover 26 candidate genes for cytochrome P450 and 17 uridine diphosphate glycosyltransferases that are involved in the production of triterpene saponin (Liao et al., 2020). As can be seen in **Supplementary Table 1**, the findings were beneficial to both the production of triterpenoid saponin and research into functional genomics.

There are several physiologically active compounds in *Lantana camara* (Lantana), including steroidal saponins, flavonoids, and glycosides. Using tools for sequencing the transcriptome, Shah et al. (2020) put together *L. camara* leaves and roots from scratch. It was found that 72,877 and 513,985 unigenes were present in leaves and roots, respectively. Of these, 229 and 943 genes were responsible for the production of phenyl-propanoic acid. As a broad-spectrum antibiotic, *Tetrastigma hemsleyanum* extract is used to treat fever and sore throats. An in-depth analysis of the metabolome and transcriptome of purple and green *T. hemsleyanum* leaves was performed by Yan et al. (2020). 4211 transcripts have been identified in the purple and green leaves, 209 metabolites have been found to be differentially expressed, and 16 chemicals have been associated with 14 transcripts implicated in the pathway for anthocyanin synthesis. The sesquiterpene lactones produced by *Saussurea lappa* have a high medicinal value. In a study conducted by Bains et al. (2019), the leaf transcriptome of *S. lappa* was sequenced to identify flavonoid and sesquiterpene-producing transcripts. Transcripts from genes implicated in alkaloid metabolism have been identified in a small number of cases. As a result of these insights, scientists will be able to learn more about how plants’ functional genomes work. Transcriptome analysis of *Arisaema heterophyllum* Blume and its leaf, tuber, and root tissues identified 47783, 43363, and 35686 unigenes, respectively, implicating genes involved in isoflavone biosynthesis. Experimental confirmation of 87 candidate genes encoding isoflavone-producing enzymes was accomplished (Wang et al., 2018). The findings of this study pave the way for further research on the pharmacological action of Arisaema. The antioxidative and anti-inflammatory properties of flavonoids, along with their application in the treatment of diseases, are illustrated in **Supplementary Table 1**.

A significant percentage of flavonoids may be found in the leaves of *Ginkgo biloba*. Wu et al. (2018) identified 37,625 unigenes from transcriptome sequencing of *G. biloba* with various flavonoid concentrations. According to the research, several potential genes are involved in the manufacture, transport, and regulation of flavonoids, according to the research. It was found that MYB transcription factor and dihydroflavonol-4-reductase, two of the fourteen flavonoid transport genes, participate in flavonoid transport. It is anticipated that the discoveries will help expand the current *G. biloba* gene database, broaden Ginkgo species research, and provide crucial information for the development of Ginkgo-related pharmaceuticals. *De novo* transcriptome sequencing of *Abrus mollis* leaves enabled analysis of flavonoid synthesis routes and associated precursors (Yuan et al. (2018)). Liu MM et al. (2018) found 99,807 unigenes in *Artemisia argyi* leaf, root, and stem tissues, including many genes that encode terpene-synthesis enzymes and transcription factors. It is anticipated that the findings will be used to investigate the molecular pathways of *A. argyi.* An analysis of *Panax ginseng* root tissues using 454-sequencing technology was conducted by Jayakodi et al. (2014). There were 17 percent difference in transcript levels between adventitious and common roots, as well as a 21 percent difference in ginsenoside-producing genes of *P. notoginseng* (Luo et al., 2011; Liu MH et al., 2015). The transcripts of *P. notoginseng* differed by 17% between adventitious and common roots, as did 21 ginsenoside-producing genes (Luo et al., 2011; Liu MH et al., 2015). Jayakodi et al., 2015 studied the transcriptomic profile of *P. ginseng* leaves, roots, and flowers and identified 107,340 unigenes, including 9,908 metabolic pathways and 270 triterpene saponin-producing genes. Among 32 genes expressed specifically in annual ginseng roots, seven genes were expressed specifically in 6-year-old ginseng roots, and 38 genes were implicated in triterpene saponin synthesis, as shown in **Supplementary Table 1**.

### The transcriptomic process

Transcriptomic approaches have been widely used to identify genes involved in plant growth and development, to identify genes that are expressed differently under abiotic stress, and to study their resistance to it. Identifying key influencing components can be helpful in plant cultivation and breeding, as well as simplifying the selection of improved varieties (Rastogi et al., 2019). Using transcriptome sequencing, Liu MM et al. (2018) studied the response of *A. argyi* leaves to cold, drought, waterlogging, and salt stress. Cold stress was the most damaging condition to the plants. Treatments with abiotic stress also reduced eugenol synthesis. The discovery of several stress-tolerance genes in *A. argyi* has enabled transgenic or polymerized plants to become stress-tolerant. According to Li et al. (2020), transcriptome sequencing was used to investigate the molecular mechanisms behind *Salvia miltiorrhiza* tissues’ responses to mild abiotic stress (drought). In total, 58,085 unigenes were discovered, of which 28,846 could be identified as such. Significant enrichment in metabolic processes and catalytic activities were found among differential transcripts in roots and leaves based on GO enrichment studies. The expression of genes that encode enzymes involved in the synthesis of phenylpropanoids and terpenoids increased in response to moderate drought stress. These findings have provided a solid foundation for further research into the process of manufacturing therapeutic components in *S. miltiorrhiza* as well as irrigation techniques for successful cultivation, as shown in **Supplementary Table 1**.

Using the third sequencing technique, Feng et al. (2019) sequenced the full-length transcriptome of *Angelica sinensis*, and differential expression sequencing was done on the wild-type transcriptome. An analysis of NGS data identified 25,463 transcripts with differential expression. There was a significant difference between transcripts in the pathway for plant-pathogen interaction and signal transduction of plant hormones. The purpose of this study is to lay out a platform for screening and developing *A. sinensis.* The transcriptome sequencing of callus tissue of *S. laniceps* was used to identify genes associated with frost resistance (Xu, 2018). In the GO enrichment investigation, 155 substances related to low temperatures, oxidative stress, and plant hormone responses were identified. Based on the KEGG enrichment analysis, several pathways were significantly enriched during low-temperature responses, including ribosomes, fatty acid metabolism, and unsaturated fatty acid biosynthesis (**Supplementary Table 1**). The findings of this study provide a framework for future research on genes associated with frost resistance in *Saussurea laniceps*.

## Discussion

In the biological sciences, transcriptome sequencing is an important sequencing technique that can be used without genomic reference sequences. Transcriptomic analysis can be used to analyze a wide range of plants and has many applications. The application of transcriptomics for obtaining genetic information about plants is growing rapidly due to its fast, high coverage, efficiency, and high throughput characteristics. This technology has been applied to mining new functional genes, analyzing secondary metabolite pathways, identifying plant developmental pathways, and obtaining helpful information for plant breeding (Li et al., 2018). Understanding secondary metabolite synthesis pathways and associated genes will benefit secondary metabolism regulatory network analysis and secondary metabolism studies in plants. Numerous plants have been analyzed by transcriptomic analysis, and the technique has been applied in many different fields. The use of transcriptomics to gather genetic information on plants is expanding as a fast, high-coverage, high-throughput, and high-efficiency analytical approach. Using the technology, researchers have been able to identify plant developmental pathways, mine novel functional genes, and analyze secondary metabolite synthesis pathways (Li et al., 2018).

Some plants produce multiple secondary metabolites to survive with biological and abiotic stresses, some of which can be used to treat a variety of human ailments. These plants are commonly referred to as plants because of their medicinal potential. Approximately 270,000 plants species have been identified globally, with less than 40,000 species having putative medical significance (Mamedov and Nazim, 2012; Tan et al., 2015). Except for a few model plants that are important research tools and sources of genomic data, most plants are largely underexplored in terms of biological information. There is a significant gap in plant genomic data due to a lack of research knowledge about the characterization of plant transcriptomes. This hinders research on important topics like the identification of significant differentially expressed genes (DEGs) and pathways related to secondary metabolite biosynthesis. In order to gather data for future plant research, plant transcriptome research should be encouraged (Navin and Hicks, 2011). In the future, transcriptome data analysis and study will assist in identifying functional genes associated with secondary metabolism pathways.

NGS techniques help us understand the transcriptome’s complexity. In several transcriptome researches on plants, NGS reads are limited by the requirement for assembly or reference genomes. The short read length of NGS technology makes it difficult to study full-length transcripts in plants. It is usually possible to investigate only the local structure of the gene and alternative splicing mechanisms for full-length transcripts. Third-generation (3G) sequencing technology is becoming more common with the advancement of sequencing techniques. Due to its relatively high sequencing cost and low throughput, third-generation sequencing is limited in its utility for transcription at this time. RNA-Seq research is mostly conducted using NGS technology, with the third generation serving as a backup. Genomic, transcriptomic, metabolomic, and proteomic technologies are also available for collaborative research. Biological functions are currently hot research topics for high-throughput sequencing, and the data will be useful for mining genes and algorithms, for fast analysis, and for showing that these topics are very useful for mining genes and algorithms.

A transcriptome profiling approach is required to identify isolated cells in a population of plants or animals, and the observations are often reflective of the number of cells predominating (Ranzoni et al., 2019). Due to cell heterogeneity, phenotypic characteristics may appear to be the same, but genetic information will vary dramatically. Consequently, transcriptome sequencing typically results in the loss of a great deal of low-abundance information (Navin and Hicks, 2011). It is predicted that single-cell transcriptome research will move into a new phase with the development of sequencing technology and the sharp decline in sequencing costs. It is possible to systematically track the dynamic changes of individual cells using single-cell transcriptome sequencing, which can effectively support the heterogeneity of single-cell gene expression that conventional sequencing ignores. We can thus gain a better understanding of cell state, genetic makeup, gene expression, and regulation, as well as develop herbal medicines.

## Conclusion

As a precursor to NGS developments, first-generation sequencing technologies and pioneering computing and bioinformatics tools generated the initial sequencing data and information within a structural and functional genomics framework. With NGS, high-throughput sequencing options are substantially cheaper, friendlier, and more flexible, allowing us to generate much more data on genomics and transcriptomics that can be used to further explore proteomics and metabolomics. A variety of NGS platforms have been released. During the first three decades of sequencing, Sanger sequencing dominated, but cost and time were major obstacles. The emergence of the second generation sequencing in 2005 and subsequent years has set the stage for breaking through the limitations of the first-generation sequencing. Sequencing by synthesis and sequencing by ligation are the two approaches proposed so far for second-generation sequencing.

There are currently more advantages to “third-generation sequencing” platforms (compared to first- and second-generation NGS platforms), including longer run times, complete transcript sequencing, and faster turnaround times. Their high mismatch rate, however, limits their use in transcriptome sequencing. On the other hand, third-generation (3G) sequencing technology can be used in conjunction with NGS technology to repair errors and offer genotyping recognition. As the cost of third-generation sequencing decreases and the accuracy of the technology improves, third-generation sequencing will become more frequently used in transcriptome research for accurate and complete transcriptome sequencing. The economic crop research model should lead to the widespread use of transcriptome sequencing in traditional plants. The majority of plant research does not rely solely on RNA-Seq at the moment. As RNA-Seq technology develops, multi-omics-related techniques will play a significant role, along with metabolomics and proteomics. Plant research would be modernized with the advancement of transcriptomic approaches, including developing metabolomics and proteomics techniques.

NGS has become a science that integrates biological information systems with big data, but many challenges remain for NGS data acquisition, analysis, storage, interpretation and integration. In order to continue producing comprehensive, high-throughput data for analysis and production, new technologies and large-scale collaboration efforts will be needed in the future. With the advent of affordable benchtop sequencers and third-generation sequencing tools, smaller laboratories and individual scientists can participate in the genomic revolution and contribute new knowledge to structural genomics and functional genomics in the life sciences.

## Author contributions

PT, DS, SM, and AS wrote the manuscript and RR edited the same for further improvement.
