# Supplementary material for: Upcoming progress of transcriptomics studies on plants: An overview
Source: Front Plant Sci. 2022 Dec 15;13:1030890. doi: 10.3389/fpls.2022.1030890 (PMC9798009; doi:10.3389/fpls.2022.1030890)
Supplement: Supplementary file 1 [file Table_1.docx]

**Supplementary Table 1: List of the next generation sequencing tools used for transcriptomics in various plants.**

| **Botanical Name** | **Plant parts used** | **NGS Tools** | **Total data generated** | **Therapeutic Potential of Plants** | **References** |
| --- | --- | --- | --- | --- | --- |
| *Terminalia arjuna* (Arjun) | Bark | Illumina NextSeq500 | 131 million reads | Used for anginal pain, congestive heart, hypertension | Srivastava et al., 2020 |
| *Aconitum*  *heterophyllum* (Atis) | Roots and  Shoots | Solexa/Illumina | Root-23.8 GB  Shoot-14.8GB | Used for diarrhea, urinary infections (UTI), and inflammation | Pal et al., 2015 |
| *Azadirachta indica* (Neem) | Fruit and Leaf | PacBio SMRT/  Illumina *HiSeq*2000 | 12 Gb | Used for leprosy, eye disorders, bloody nose | Rajakani et al., 2014 |
| *Andrographis paniculata* (Kalmegh) | Leaf and root | Illumina *HiSeq*2000 | Leaf-69,011 and root- 64,244 | Used in cancer, diabetes, and high blood pressure | Cherukupalli et al., 2016 |
| *Arabidopsis lyrata* (Lyrate rockcress) | Whole plant | Sanger | 96% data obtained | Specially leaves are used for vegetable and salads | Rawat et al., 2015 |
| *Arabidopsis thaliana* (Thale cress) | Whole plant | Sanger, 454, Illumina | 96% data obtained | Provide specific defense mechanisms of plant-pathogen resistance | Desikan et al., 2001 |
| *Artemisia annua* (Sweet Wormwood) | Trichome | Illumna *HiSeq* 4000 | 266.7 million | Malaria and related symptomes (fever, chills) | Wang et al., 2009 |
| *Artemisia tridentate* (Big sagebrush) | Leaf | Roche 454, Illumina | 823,392 sequence reads and 695 SNP position | Use for firewood | Prabin., 2011 |
| *Asparagus racemosus* (Satawar) | Leaf and root | Illumina GAII Analyzer/454 GS FLX/5500 SOLiD | Leaf- 6.79 Gb and root- 7.43 Gb | Used in stomach related problems | Upadhyay et al., 2014 |
| *Azadirachta indica* (Neem) | leaf and fruit | Roach 454 | Leaf-273.8 Mb  Fruit-220 Mb | For leprosy, eye, stomach problems, and bloody nose, intestinal worms | Krishnan et al., 2011 |
| *Benicasa hispida* (Wax Gourd) | Leaf, flowers, fruits, and stems | Illumina | 4 Gb | It is used laxative, diuretic, tonic, aphrodisiac, cardiotonic, and menstrual disorders | Jiang et al., 2013 |
| Ramie (*Boehmeria nivea*)(Chinese Grass) | Leaf and root | Illumina *HiSeq* 2000 | 40.2 and 62.8 million reads | Threatened abortions, colic of pregnancy, and leucorrhoea, impetigo | Touming et al., 2014 |
| *Brachypodium*  *Distachyon* (Purple false brome) | Leaf and stem | Sanger | 90 % reads | Experimental model organism for understanding | Priest., 2014 |
| *Bupleurum chinense* (Bupleurum) | Roots | Roche 454 | 195, 088 high-quality reads | Treating fatty liver disease | Sui et al., 2011 |
| *Camelina sativa* (False flax) | Flower and Leaf | Illumina | 98 % reads | Used edible oil and biodiesel form | Kagale et al., 2014 |
| *Cyanotis arachnoidea* (Grass of the Dew) | Leaf and root | Illumina *HiSeq*2500 | 86.5 million clean reads | Cure the rheumatic infections | Xiu et al., 2018 |
| *Camptotheca acuminata* (Happy Tree) | Leaves | Illumina *HiSeq*2500 | Leaf-45,368,044 and root-41,095,316 clean reads | Particularly used for cancer patients | Sun et al., 2011 |
| *Cananga odorata* (Kenanga**)** | Flower | Illumina | 110 million reads | Used in malaria, stomach ailments, and asthma | Jin et al., 2015 |
| *Cassia angustifolia* (Alexandrian senna) | Young and mature leaf | Illumina | 6.34 Gb | Used for laxative | Reddy et al., 2015 |
| *Catharanthus roseus* (Bright eyes) | Leaf, roots, Cell suspensions, shoot | Illumina *Hiseq* 2000 | 32,891,528,340 nt bases | Use for relieving muscle pain, depression of the central nervous system | Verma et al., 2014 |
| *Centella asiatica* (Gotu Kola) | Leaf | Illumina | 46,171,131 reads | Used to heal wounds, improve mental clarity, and treat skin conditions | Sangwan et al., 2013 |
| *Chelidonium majus* (Celandine) | Stem | Illumina *HiSeq* 2000 | 188.98 million reads | Used to improve eyesight and whooping cough, asthma, and jaundice | Nawrot et al., 2016 |
| *Chlorophytum borivilianum* (Safed musli) | Leaf | Illumina *HiSeq* 2000 | 33,963,356 high-quality reads | Leaves and roots are used for vegetable and health tonic form | Kalra et al., 2013 |
| *Cicer arietinum* (Chickpea) | Root and shoot | Roche 454 GS | 1.38 million reads | Antioxidant, antidiabetic, anti-inflammatory, antimicrobial | Garg et al., 2011 |
| *Cistanche deserticola* (Desert-broomrape) | Fleshy stem | Illumina *HiSeq*2000 | 80 million reads | Use in hormone regulation, antioxidative, anti-apoptotic, anti-nociceptive, anti-inflammatory, activities and the promotion of bone formation | Li et al., 2015 |
| *Conyza canadensis* (Horseweed) | Seed | Illumina *HiSeq* 2500 | 411 962 raw reads | Treatment of diarrhoea, dysentery, and internal haemorrhages | Peng et al., 2010 |
| *Costus pictus*  (Painted Spiral Ginger) | Leaf | Illumina | 3.2Gb | For its tonic, stimulant, and antiseptic properties | Annadurai et al., 2012 |
| *Dendrobium officinale* (Orchid) | Stem | Illumina | 86 million reads | Nourishing the stomach, enhancing production of body fluids or nourishing Yin | Guo et al., 2013 |
| *Dianthus caryophyllus* (Carnation) | Flower bud, flower | Roche 454 | 1,162,126 high-quality reads | To treat coronary and nervous disorders | Tanase et al., 2012 |
| *Digitalis purpurea* (Foxglove) | Leaf, stem, flower, root | Roche 454 | 66103 high-quality reads | Used for heart related problems | Wu et al., 2012 |
| *Epimedium sagittatum* (Horny goat weed) | Leaf | Roche 454 | 55 million | Used for hay fever, sexual dysfunctions | Zeng et al., 2010 |
| *Euphorbia fischeriana* (S*purge)* | Roots | Illumina *HiSeq*2000 | 9,6481,893 raw reads | Dyspepsia, abdominal distension, abdominal pain, cough, scabies, tuberculosis of lymph nodes | Barrero et al., 2011 |
| *Fraxinus americana*  (White ash) | Phloem plug | Roche 454 | 58,673 high quality reads | Used for starting fires and barbecues, and is usable for maintaining a fire | Bai et al., 2011 |
| *Glycine max* (Soya bean) | Seed | Illumina *HiSeq*™ 2000 | 88,643 transcripts | Salad oil and for manufacture of margarine | Severin et al., 2010 |
| *Gastrodia elata* (Tianma) | Vegetative propagation corms, Juvenile tuber | Illumina *HiSeq*™ 2000 | 179.1 Gb raw sequencing reads | It is used for headache, dizziness, spasm, epilepsy, stoke, etc. | Tsai et al., 2016 |
| *Gynostemma pentaphyllum* (Jiaogulan) | Root, leaves | Roche 454 | 352,999,296 original reads | High cholesterol, high blood pressure, and improving heart function | Subramaniyam et al., 2011 |
| *Ginkgo biloba* | root, stem, leaf, microstrobilus, ovulate strobilus, immature fruit, and mature fruit | PacBio Iso-Seq | 23.36 Gb of clean reads | Alzheimer's disease, Anxiety, Asthma, Bronchitis | Ye et al., 2019 |
| *Huperzia serrate* (Toothed clubmoss) | Leaves | Illumina *HiSeq*4000 | 40.1 Gb of clean data | Dietary supplement to improve brain function | Luo et al., 2010 |
| *Jatropha curcas* (Barbados nut) | Vegetative | Illumina *HiSeq* 2500 | 64,235 reads | Bacterial and fungal infections or febrile diseases, muscle pain or jaundice | Gangwar., 2020 |
| *Litsea cubeba*  (Aromatic litsea) | Flower, leaf, and fruit | Illumina | 160.88 Gb | Antibacterial, Anti-inflammatory, and improve heart health | Han et al., 2013 |
| *Macleaya cordata* (Plume poppy) | Roots, leaves and fruits | Illumina *HiSeq*2000 | 6.02 Gb | Analgesic, antioedema, carminative, depurative and diuretic | Zeng et al., 2013 |
| *Piper nigrum* (Black pepper) | Fruit, root | Illumina, SOLiD | 13.27G of raw sequence data | Kill fungi and parasites, and help the body absorb some drugs | Sheila et al., 2012 |
| *Trachyspermum ammi* (Ajwain) | Different inflorescence tissue | *HiSeq*2000 platform | 43,056,120 and 42,260,830 of high-quality reads | Used for abdominal related problems, and asthma | Howyzeh et al., 2018 |
| *Withania somnifera* (Ashwagandha) | Root and leaf | Roche 454-GS FLX | 249 million high-quality reads | Used for hypertension, stress, asthma, arthritic diseases | Gupta et al., 2013 |
| *Xanthium strumarium* (Rough cocklebur) | Leaf | Illumina | 157 million clean reads | Used for laxative, fattening, digestive, antipyretic, and diuretic | Fan et al., 2015 |
| *Triticum aestivum* (Wheat) | Seeds and Stem | Illumina *HiSeq* 2000 | 97,155,472 reads | Flour and breadstuffs | Liu et al., 2016 |
| *Oryza sativa* (Rice) | Seeds and Stem | Illumina *HiSeq* 2000 | 39.73 Gb | Ash for discharges and wounds, sapraemia in Malaria infusion of straw for dysentery, gout, and rheumatism | Sun et al., 2019 |
| *Zea mays* (Maize) | Seeds | Illumina *HiSeq* 2000 | 8~10 million raw reads | Used for bladder infections, kidney stones, and bedwetting | Wu et al., 2017 |
| *Hordeum vulgare* (Barley) | Seeds | Illumina *HiSeq* 2000 | 208 million clean reads | Used for soups, stews | Tombuloglu et al., 2015 |
| *Sorghum bicolor* (Sorghum) | Seeds | Illumina *HiSeq* 2000 | 31 to 39 million quality reads | Used for food, feed | Dugas et al., 2011 |
| *Pennisetum glaucum* (Pearl millet) | Seeds | Illumina *Hiseq* | 139.1 million reads | Used for making gluten-free cereal-based products | Dudhate et al., 2018 |
| *Eleusine coracana* (Ragi) | Seeds | Illumina NextSeq 500 | 44,861,506 million reads | Remedy for leprosy, liver disease, measles, pleurisy, pneumonia, and smallpox | Parvathi et al., 2019 |
| *Amorphophallus bulbifer* (Devil's Tongue) | Leaf | Illumina | 54,986,020 reads and 52,334,098 reads | Used for tumour-suppression, phlegm liquefaction, and blood stasis alleviation | Zheng et al., 2013 |
| *Mangifera Indica* (Mango) | Fruit | Illumina *HiSeq*™ 2500 | 400 million reads | Astringent, diaphoretic, stomachic, anaemia, asthma, bronchitis, cough, hypertension, insomnia, rheumatism, toothache, leucorrhoea | Tafolla-Arellano et al., 2017 |
| *Psidium guajava*  (Guava) | Fruit | Illumina *HiSeq*2500 | 20 million raw reads | Inflammation, diabetes, hypertension, caries, wounds, lung diseases, and ulcers | Mittal et al., 2020 |
| *Musa paradisiaca* (Banana) | Fruit | Illumina *HiSeq* 2500 | 89.30% of total reads | Used for low levels of potassium in the blood (hypokalemia), constipation, and diarrhea | Kaur et al., 2021 |
| *Actinidia chinensis* (kiwifruits) | Fruit | Illumina | 305.24 Gb | Asthma, constipation, high blood pressure | Hill et al., 2015 |
| *Malus domestica* (Apple) | Fruit | Illumina *HiSeq* 2000 | 327.5 million reads | Rich in fibre, vitamins, and minerals | El-Sharkawy et al.,  2015 |
| *Ficus carica* (Fig) | Fruit | Illumina *HiSeq* 4000 | 32,429,280  clean reads | Constipation, diarrhea, diabetes, high cholesterol, and skin conditions | Wang et al., 2017 |
| *Cistus creticus* (Cretan Rockrose) | Trichomes | Sanger | - | Used internally in the treatment of catarrh and diarrhoea and as an emmenagogue | Vasiliki et al., 2008 |
| *Citrullus lanatus* (Watermelon) | Fruit flesh | Roche 454 | 80.6 Gb | Used as purgative and emetic in high dose, vermifuge, demulcent, diuretic, and tonic | Zhang et al., 2015 |
| *Citrus clementine* (orange) | Leaf | Sanger | 140.7 Gb | Rich in antioxidants, which help reduce inflammation | Marco et al., 2012 |
| *Morus alba* (Mulberry) | Fruit | Illumina *HiSeq*™ 2500 | 33.77–74.56 million reads | Used for heart related problem | Huang et al., 2020 |
| *Ananas comosus* (Pineapple) | Fruit | Illumina *HiSeq*™ 2500 | 149 GB | Rich in vitamin C and anti-inflammatory and pain-relieving properties | Qi et al., 2018 |
| *Vitis vinifera* (Grapea) | Fruit | Illumina NovaSeq 6000 | 0.83 billion clean reads | Nutrients, antioxidants, and powerful plant compounds | Crystal et al., 2012 |
| *Punica granatum* (Pomegranate) | Fruit | Illumina *HiSeq*X Ten | 106.27 Gb | Effective in preventing prostate, breast, lung, and colon cancers | Fang- fang et al., 2021 |
| *Citrullus lanatus* (Watermelon) | Fruit | Illumina *HiSeq* 2000 | 321.8 million high quality | Used as purgative and emetic in high dose, vermifuge, demulcent, diuretic, and tonic | Guo., 2015 |
| *Aegle marmelos* (Bel) | Fruit | Illumina *HiSeq* 2500 | 49.58 million raw reads | Dropsy, bleeding piles, dysentery, diarrhea | Kaushik et al., 2019 |
| *Tectona grandis* (Teak) | Wood | Illumina *MiSeq*^TM^ 2000 | 3,778,316 reads | Used for boat building, exterior construction, veneer, furniture, carving, turnings, and other small wood projects | Diningrat et al., 2015 |
| *Dalbergia sissoo* (Sheesham) | Wood | Illumina *HiSeq*4000 | 71 to 180 million paired end reads | Used for the treatment of obesity, vitiligo, fever, non-healing wounds, ulcers, intestinal parasites | Tin et al., 2020 |
| *Hevea brasiliensis* (rubber tree) | Leaf and Latex | Illumina *HiSeq* 2000 | 4.00 G | Cured rubber used for all types of rubber products | Liu et al., 2015 |
| *Corchorus olitorius*  (Jute) | Stem and ribbon | Illumina *HiSeq*X Ten | 608,395,184 raw reads | Fabrics for packaging a wide range of agricultural | Zemao et al., 2017 |
| *Gossypium hirsutum* (Cotton) | Seeds and bolls | Illumina *HiSeq*4000 | 136.49 Gb | To make several textile products and oil. | Mingge et al., 2019 |
| *Linum usitatissimum* (Flax) | Seeds | Illumina NextSeq 500 | 16 million reads | Flax fibres are used to make linen and rope | Alexey et al., 2020 |
| *Bambusa vulgaris* (Bamboo) | Stem, seeds | Illumina | 2.075 Gb | Flooring, roofing designing, and, furniture | Biswas et al., 2016 |
| *Cocos nucifera* (Coconut) | Seed and fruit | Illumina *HiSeq*™ 2000 | 54.9 million reads | Used to treat diarrhoea | Fan et al., 2013 |
| *Agave sisalana* (Sisal) | leaves | Illumina *HiSeq*2500 | 276,845,790 reads | Used for rope and twine | Muhammad et al., 2019 |
| *Cannabis sativa* (Hemp) | seeds, stalks, roots and leaves | Illumina *HiSeq*3000 | 30 million reads | Rope, textiles, clothing, shoes, food, paper, bioplastics, insulation, and biofuel | Braich et al., 2019 |
| *Rosa indica* (Rose) | Flower | Illumina *HiSeq* 4000 | 168.97 GB | Ornamental plants grown for their flowers in the garden and sometimes indoors. | Tian et al., 2018 |
| *Hibiscus-roja-sinensis* (Gudhal) | Flower | 454 sequencing | 82.2Mb | Used in the treatment of excessive and painful menstruation, cystitis, venereal diseases, feverish illnesses, bronchial catarrh, coughs and to promote hair growth | Alice et al., 2016 |
| *Fritillaria cirrhosa* (Yellow Himalayan fritillary) | Bulb | Illumina | 150 million clean reads | Coughs, bronchitis, pneumonia, asthma, feverish illnesses, abscesses | Zhao et al., 2018 |
| *Tagetus erectus* (Marigold) | Flower | Illumina *HiSeq* 2000 | 21,795,753 clean reads | Used for digestive tract problems including poor appetite, gas, stomach pain, colic, intestinal worms, and dysentery | Ai et al., 2016 |
| *Jasminum sambac* (Mogra) | Flower | Illumina *HiSeq*  2000 | 42 million reads | Used for anti-depressant, antiseptic, cicatrisant, aphrodisiac, and expectorant | Yong- Hua et al., 2015 |
| *Jasminium Dumort* (Jasmine/Chameli) | Flower | Illumina *HiSeq*  2500 | 42 million  reads | Used to make jasmine tea, which often has a base of green tea or white tea | Li et al., 2015 |
| *Chrysanthemum nankingense* (East Asia) | Stems and leaves | Illumina *HiSeq*^TM^ 2000 | 7.47 million | Used for high blood pressure, type 2 diabetes, fever, and cold | Wang et al., 2013 |
| *Nelumbo nucifera* (Lotus) | Flower | Illumina *HiSeq*^TM^ 2000 | 348 million high-quality reads | Bleeding, cough, fever, liver and stomach problems, and other conditions | Mei et al., 2015 |
| *Lilium* (Lili) | Flower | Illumina *HiSeq*2500 | 29.24 Gb | Used in herbaceous borders, and shrub plantings, and as patio plants | Hu et al., 2017 |
| *Carthamus tinctorius* (Sunflower) | Flower | Illumina | 56,960,100 clean reads | Cultivated for vegetable oil and substitute for saffron | Lulin et al., 2012 |
| *Amsonia hubrichtii* (Hubricht's Bluestar) | Leaf | Roache 454-  Illumina GA- | Roache 454 48.1% (715,432) and Illumina 63.2% (75,544,560 reads) | Butterfly Nectar Plant | Xiao et al., 2013 |
| *Abelmoschus esculentus* (Bhindi) | Fruits | Illumina *HiSeq* 2500 | 751,403,932 reads | Promotes Heart Health, Regulates Blood Sugar, Immunity, prevent anaemia, weight reduces, and beneficial in pregnancy | Shi et al., 2020 |
| *Allium cepa* (Onion) | Bulb, leaves,  Root | Roche 454 | 16.3 Gb | Ailments like headaches, heart disease and mouth sores | Han et al., 2016 |
| *Allium sativum* (Garlic) | Bulb | Illumina | 15.9 Gb | Fight inflammation, reduce cholesterol levels, and protect against chronic disease | Mayer., 2015 |
| *Raphanus sativus* (Radish) | Root and leaf | Illumina *HiSeq*™ 2000 | 54.64 million clean reads | Consumed mainly as a salted vegetable and are also eaten fresh as grated radish, garnish, and salad | Nie et al., 2015 |
| *Daucus carota* (Carrot) | Root | Illumina *HiSeq*™ 2000 | 41.74-46.30 million clean reads | Used to prevent cancer, and for digestive health, obesity, other nutrient deficiencies | Geng et al., 2020 |
| *Capsicum annuum* (Chilli) | Berry-fruit | Illumina *HiSeq* 4000 | 39.86 million reads | Used in cooking, pickles, and chutney | Jie., 2016 |
| *Zingiber officinalis* (Ginger) | Rhizome | Illumina *HiSeq*™ 2500 | 19.22 GB | As a food flavouring and medicine | Yusong et al., 2017 |
| *Solanum tuberosum* (Potato) | Tuber | Illumina *HiSeq*X10 | 77.89 million paired end reads | Potatoes are a good source of fibre | Qing et al., 2020 |
| *Solanum lycopersicum* (Tomato) | Fruits | Illumina *HiSeq*™ 2500 | 564.8 million raw reads | Used for first aid treatment for burns, scalds, and sunburn | Zhan et al., 2018 |
| *Lagenaria siceraria* (Loki) | Fruits | Illumina *HiSeq* 2000 | 41,831,589 high quality reads | Used for diabetes mellitus, liver diseases, weight loss | Zhang et al., 2018 |
| *Luffa acutangula* (Toari) | Fruits | Illumina NovaSeq 6000 | 19.51 Gb | Reducing inflammation, weight loss, controlling diabetes | Sun et al., 2022 |
| *Momordica charantia* (Karela) | Fruits | Illumina Genome Analyzer II | 15.06 GB | Gonorrhoea, measles, chicken pox, scabies, and malaria | Shukla et al., 2015 |
| *Cucurbita pepo* (Pumpkin) | Fruits | Illumina *HiSeq*™ 2000 | 17 Gb of clean paired end reads | Highly Nutritious and Particularly Rich in Vitamin A,  High Antioxidant Content, and Vitamin A, Lutein and Zeaxanthin | Guo et al., 2018 |
| *Cucumis sativus* (Kheera) | Fruits | Illumina *HiSeq* 2500 | 7.7-41.4 million raw reads | Detoxifies, keep brain health, relieves constipation, keeps body cool | Xiaolei et al., 2018 |
| *Artocarpus heterophyllus*  (Jackfruit) | Fruits | Illumina *HiSeq* 2500 | 30 million clean reads | As food or as medicine | Hu et al., 2016 |
| *Capsicum annuum* (Chili Pepper) | Fruits | Illumina *HiSeq* 4000 | 39.86 million reads | Used for upsetting stomach, toothache, poor circulation, fever, hyperlipidaemia, and heart disease prevention | Ashrafi et al., 2012 |
| *Spinacia oleracea* (Palak) | Leaves | Illumina *HiSeq* 2500 | ∼100 Gb | Beneficial for skin, hair, and bone health. They also provide protein, iron, vitamins, and minerals | Chenxi et al., 2017 |
| *Trigonella foenum-graecum* (Methi) | Leaves | SOLiD 4 | 42 million high quality reads | Help in maintaining body weight or weight loss | Vaidya et al., 2013 |
| *Chenopodium album* (Bathua) | Leaf | Illumina *HiSeq*2000 | 6,991,000 Bp | Improve antioxidant properties and immunity, and rich in iron and potassium | Su- Young et al., 2017 |
| *Coriandrum sativum* (Dhaniya) | Stem and leaf | Illumina Next*Seq*500 | 43,746,120  and 49,163,108 high-quality reads | Fragrant, antioxidant-rich herb | Choudhary et al., 2019 |
| *Amorphophallus konjac* (Devil's tongue) | Leaf | Illumina | 54,986,020 reads and 52,334,098 | Food source and as a traditional medicine | Gille., 2011 |
| *Ipomoea batatas* (Sweet potato) | Root | Illumina | ~208 and ~200 million reads | Diabetes, hypertension, dysentery, constipation, fatigue, arthritis, rheumatoid diseases, hydrocephaly, meningitis, kidney ailments, and inflammations | Wang et al., 2010 |
| *Beta vulgaris* (Beet root) | Root and leaf | Roche 454, Illumina *HiSeq*2000 and Sanger | 10.52 Gb | Improved blood flow, lower blood pressure, and increased exercise performance | Mutasa-Gottgens et al., 2012 |

**References**

- Srivastava, G., Garg, A., Misra, C. R., Singh, C., Ghosh, S. (2020). Transcriptome analysis and functional characterization of oxidosqualene cyclases of the Arjuna triterpene saponin pathway, *Plant Sci, 292*:110382. doi: 10.1016/j.plantsci.2019.110382.
- Pal, T., Malhotra, N., Chanumolu, S. K., Chauhan, R. S. (2015). Next-generation sequencing (NGS) transcriptomes reveal association of multiple genes and pathways contributing to secondary metabolites accumulation in tuberous roots of *Aconitum heterophyllum* Wall, *Planta; 242*(1):239-58. doi: 10.1007/s00425-015-2304-6.
- Rajakani, R., Narnoliya, L., Sangwan, N. S., Sangwan, R. S., Gupta, V. (2014). Subtractive transcriptomes of fruit and leaf reveal differential representation of transcripts in *Azadirachta indica*. *Tree Genetics & Genomes.;10*:1331-1351. doi: 10.1007/s11295-014-0764-7.
- Cherukupalli, N., Divate, M., Mittapelli, S. R., Khareedu, V. R., Vudem, D. R. (2016). *De novo* assembly of leaf transcriptome in the medicinal plant *Andrographis paniculata*. *Front Plant Sci.; 7*:1203.
- Rawat, V., Abdelsamad, A., Pietzenuk, B., Seymour, D. K., Koenig, D., Weigel, D., Pecinka, A., Schneeberger, K. (2015). Improving the annotation of *Arabidopsis lyrata* using RNA-Seq data. *PLoS ONE 10*: e0137391.
- Desikan, R., Mackerness, S., Hancock, J. T., Neill, S. J. (2001). Regulation of the *Arabidopsis* transcriptome by oxidative stress. *Plant Physiol. 127*: 159-172. https://doi.org/10.1104/pp.127.1.159.
- Wang Z, Gerstein M, Snyder M. RNA-Seq: a revolutionary tool for transcriptomics. Nature reviews. Genetics. 2009;10:57–63.
- Prabin, B. (2011). Transcriptome Sequencing, Characterization, and Polymorphism Detectio/n in Subspecies of Big Sagebrush (Artemisa tridentata) Department of Plant and Wildlife Sciences, Brigham Young University.
- Upadhyay, S., Phukan, J. U., Mishra, S., Shukla, R. K. (2014). *De novo* leaf and root transcriptome analysis identified novel genes involved in Steroidal sapogenin biosynthesis in *Asparagus racemosus*, *BMC Genomics*.; 15(1): 746. doi: 10.1186/1471-2164-15-746.
- Krishnan, N. M., Pattnaik, S. A., Deepak, A. K., Hariharan, A. K., Gaur, P. (2011). *De novo* sequencing and assembly of *Azadirachta indica* fruit transcriptome. *Curr Sci (India) 101*: 9.
- Jiang, B., Xie, D., Liu, W., Peng, Q., He, X. (2013). *De Novo* Assembly and Characterization of the Transcriptome, and Development of SSR Markers in Wax Gourd (*Benicasa hispida*). *PLoS ONE 8*(8): e71054. https://doi.org/10.1371/journal.pone.0071054.
- Touming, L., Shouwei, T., Siyuan, Z., Qingming, T., Xia, Z. (2014). Transcriptome comparison reveals the patterns of selection in domesticated and wild ramie (*Boehmeria nivea* L. Gaud), *Plant Molecular Biology 86*(1-2), DOI:10.1007/s11103-014-0214-9.
- Priest, H. D. (2014). Analysis of Global Gene Expression in *Brachypodium distachyon* Reveals Extensive Network Plasticity in Response to Abiotic Stress. *PLoS ONE 9,* e87499.
- Sui, C., Zhang, J., Wei, J., Chen, S., Li, Y., Xu, J., Jin, Y., Xie, C., Gao, Z., Chen, H., Yang, C., Zhang, Z., Xu, Y. (2011). Transcriptome analysis of *Bupleurum chinense* focusing on genes involved in the biosynthesis of saikosaponins, *BMC Genomics. 2*; 12:539. doi: 10.1186/1471-2164-12-539.
- Kagale, S., Koh, C., Nixon, J., Bollina, V., Clarke, E., Tuteja, R., Spillane, C., Robinson, S. J., Links, M. G., Clarke, C., Higgins, E. E., Huebert, T., Sharpe, A. G., Parkin, I. A. (2014). The emerging biofuel crop Camelina sativa retains a highly undiffererentiated hexaploid genome structure. *Nat. Commun., 5*, p. 3706, 10.1038/ncomms4706.
- Xiu, Y. L., Jing, X., Jian, W. W., Li, P. Z. (2018). Comparative Transcriptome Analysis Identifies Genes Putatively Involved in 20-Hydroxyecdysone Biosynthesis in *Cyanotis arachnoidea*, *Int J Mol Sci. 27*;19(7):1885. doi: 10.3390/ijms19071885.
- Sun, Y., Luo, H., Li, Y. (2011). Pyrosequencing of the *Camptotheca acuminata* transcriptome reveals putative genes involved in camptothecin biosynthesis and transport. *BMC Genomics; 12*(1):533.
- Jin, J., Kim, J. M., Dhandapani, S., Tjhang. J. G., Yin, J. L., Wong, L., Sarojam, R., Chua, N. H., Jang, I. C. (2015). The floral transcriptome of ylang (Cananga odorata var. fruticosa) uncovers biosynthetic pathways for volatile organic compounds and a multifunctional and novel sesquiterpene synthase, J Exp Bot.;66(13):3959-75. doi: 10.1093/jxb/erv196.
- Reddy, N. R., Mehta, R. H., Soni, P. H., Makasana, J., Gajbhiye, N. A., Ponnuchamy, M. (2015). Next Generation Sequencing and Transcriptome Analysis Predicts Biosynthetic Pathway of Sennosides from Senna (*Cassia angustifolia* Vahl.), a Non-Model Plant with Potent Laxative Properties. *PLoS ONE 10*(6): e0129422. https://doi.org/10.1371/journal.pone.0129422.
- Verma, M., Ghangal, R., Sharma, R., Sinha, A. K., Jain, M. (2014). Transcriptome Analysis of *Catharanthus roseus* for Gene Discovery and Expression Profiling. *PLoS ONE 9*(7): e103583. https://doi.org/10.1371/journal.pone.0103583.
- Sangwan, R. S., Tripathi, S., Singh, J., Narnoliya, L. K., Sangwan, N. S. (2013). *De novo* sequencing and assembly of *Centella asiatica* leaf transcriptome for mapping of structural, functional, and regulatory genes with special reference to secondary metabolism. *Gene, 525*, 58-6.
- Nawrot, R., Barylski, J., Lippmann, R., Altschmied, L., Mock, H. P. (2016). Combination of transcriptomic and proteomic approaches helps to unravel the protein composition of *Chelidonium majus* L. milky sap, *Planta 244*; 1064.
- Kalra, S., Puniya, B. L., Kulshreshtha, D., Kumar, S., Kaur, J., Ramachandran, S., Singh, K. (2013). *De novo* transcriptome sequencing reveals important molecular networks and metabolic pathways of the plant, *Chlorophytum borivilianum*. *PLoS One. 23*;8(12): e83336.
- Garg, R., Patel, R. K., Jhanwar, S., Priya, P., Bhattacharjee, A., Yadav, G., Bhatia, S., Chattopadhyay, D., Tyagi, A. K., Jain, M. (2011). Gene Discovery and Tissue-Specific Transcriptome Analysis in Chickpea with Massively Parallel Pyrosequencing and Web Resource Development, *Plant Physiology, 56*, 1661-1678, https://doi.org/10.1104/pp.111.178616.
- Li, Y., Wang, X., Chen, T., Yao, F., Li, C., Tang, Q., Sun, M., Sun, G., Hu, S., Yu, J., Song, S. (2015). RNA-Seq Based *De Novo* Transcriptome Assembly and Gene Discovery of *Cistanche deserticola* Fleshy Stem, *PLoS One.; 10*(5): e0125722. doi: 10.1371/journal.pone.0125722.
- Peng, Y., Abercrombie, L. G., Yuan, J. S., Riggins, C. W., Sammons, R. D., Stewart, N. C. (2010). Characterization of the horseweed (*Conyza canadensis*) transcriptome using GS-FLX 454 pyrosequencing and its application for expression analysis of candidate non-target herbicide resistance genes, *Pest Management Science 66*, 10 p. 1053-1062, https://doi.org/10.1002/ps.2004.
- Annadurai, R. S., Jayakumar, V., Mugasimangalam, R. C., Katta, M., Anand, S., Gopinathan, S., Sarma, S. P., Fernandes, S. J., Mullapudi, N., Murugesan, S., Rao, S. N. (2012). Next generation sequencing and *de novo* transcriptome analysis of *Costus pictus* D. Don, a non-model plant with potent anti-diabetic properties, *BMC Genomics 13*: 663.
- Guo, X., Li, Y., Li, C., Luo, H., Wang, L., Qian, J. (2013). Analysis of the *Dendrobium officinale* transcriptome reveals putative alkaloid biosynthetic genes and genetic markers. *Gene 527*, 131-138. doi: 10.1016/j.gene.2013.05.073.
- Tanase, K., Nishitani, C., Hirakawa, H., Isobe, S., Tabata, S., Ohmiya, A., Onozaki, T. (2012). Transcriptome analysis of carnation (*Dianthus caryophyllus* L.) based on next-generation sequencing technology, *BMC Genomics 13*: 292.
- Wu, B., Li, Y., Yan, H., Ma, Y., Luo, H., Yuan, L., Chen, S., Lu, S. (2012). Comprehensive transcriptome analysis reveals novel genes involved in cardiac glycoside biosynthesis and mlncRNAs associated with secondary metabolism and stress response in *Digitalis purpurea*, *BMC Genomics 13*: 15.
- Zeng, S., Xiao, G., Guo, J., Fei, Z., Xu, Y., Roe, B. A., Wang, Y. (2010). Development of a EST dataset and characterization of EST-SSRs in a traditional Chinese medicinal plant, *Epimedium sagittatum* (Sieb. Et Zucc.) Maxim, *BMC Genomics 11*: 94.
- Barrero, R. A., Chapman, B., Yang, Y., Moolhuijzen, P., Keeble-Gagnere, G., Zhang, N., Tang, Q., Bellaqrd, M. I. Qiu, D. (2011). *De novo* assembly of *Euphorbia fischeriana* root transcriptome identifies prostratin pathway related genes. *BMC Genom.12*:600-613. doi: 10.1186/1471-2164-12-600.
- Bai, X., Rivera-Vega, L., Mamidala, P., Bonello, P., Herms, D. A., Mittapalli, O. (2011). Transcriptomic Signatures of Ash (*Fraxinus* spp.) Phloem. *PLoS ONE 6*(1): e16368. https://doi.org/10.1371/journal.pone.0016368.
- Severin, A. J., Woody, J. L., Bolon, Y. T., Joseph, B., Diers, B. W., Farmer, A. D., Muehlbauer, G. J., Nelson, R. T., Grant, D., Specht, J. E., Graham, M. A., Cannon, S. B., May, G. D., Vance, C. P., Shoemaker, R. C. (2010). RNA-Seq Atlas of *Glycine max*: A guide to the soybean transcriptome, *BMC Plant Biology* *10*: 160.
- Tsai, C. C., Wu, K. M., Chiang, T. Y., Huang, C. Y., Chou, C. H., Li, S. J., Chiang, Y. C. (2016). Comparative transcriptome analysis of *Gastrodia elata* (*Orchidaceae*) in response to fungus symbiosis to identify gastrodin biosynthesis-related genes, *BMC Genomics 17*: 212.
- Subramaniyam, S., Mathiyalagan, R., Gyo, I. J., Bum-Soo, L., Sungyoung, L., Chun, Y. D. (2011). “Transcriptome profiling and *insilico* analysis of *Gynostemma pentaphyllum* using a next generation sequencer,” *Plant Cell Reports, 30*, 11, 2075-2083.
- Ye, J., Cheng, S., Zhou, X., Chen, Z., Kim, S.U., Tan, J., Zheng, J., Xu, F., Zhang, W., Liao, Y. and Zhu, Y. (2019). A global survey of full-length transcriptome of *Ginkgo biloba* reveals transcript variants involved in flavonoid biosynthesis. *Industrial Crops and Products*, *139*, 111547.
- Luo, H. M., Sun, C., Li, Y., Wu, Q., Song, J. Y., Wang, D. L., Jia, X. C., Li, R. T., Chen, S. L. (2010). Analysis of expressed sequence tags from the *Huperzia serrata* leaf for gene discovery in the areas of secondary metabolite biosynthesis and development regulation, *Physiol. Plant., 139*.
- Gangwar, M. (2020). Molecular Mechanisms of the Floral Biology of *Jatropha curcas*: Opportunities and Challenges as an Energy Crop, *Front Plant Sci. 9*; 11:609. doi: 10.3389/fpls.2020.00609.
- Han, X. J., Wang, Y. D., Chen, Y. C., Lin, L. Y., Wu, Q. K. (2013). Transcriptome Sequencing and Expression Analysis of Terpenoid Biosynthesis Genes in *Litsea cubeba*. *PLoS ONE 8*(10): e76890. https://doi.org/10.1371/journal.pone.0076890.
- Zeng, J., Liu, Y., Liu, W., Liu, X., Liu, F., Huang, P. (2013). Integration of Transcriptome, Proteome and Metabolism Data Reveals the Alkaloids Biosynthesis in *Macleaya cordata* and *Macleaya microcarpa*. *PLoS ONE 8*(1): e53409. <https://doi.org/10.1371/journal.pone.0053409>.
- Sheila, M. C., Pinheiro, D. G., Moreira, E. C., Rodrigues, S. M., Poltronieri, M. C., Lemos, O. F., Silva, I. T., Ramos, R. T., Silva, A., Schneider, H., Silva, W. A., Sampaio, I., Darnet, S. (2012). High-throughput sequencing of black pepper root transcriptome, *BMC Plant Biology 12*: 168.
- Howyzeh, M. S., Noori, S., Shariati, J. V., Amiripour, M. (2018). Comparative transcriptome analysis to identify putative genes involved in thymol biosynthesis pathway in medicinal plant *Trachyspermum ammi* L., *Scientific Reports 8*: 13405.
- Gupta, P. (2013). *De novo* assembly, functional annotation, and comparative analysis of *Withania somnifera* leaf and root transcriptomes to identify putative genes involved in the with anolides biosynthesis. *PLoS ONE 8*, e62714.
- Fan, R., Li, Y., Li, C., Zhang, Y., Yang, Z. M. (2015). Differential microRNA Analysis of Glandular Trichomes and Young Leaves in *Xanthium strumarium* L. Reveals Their Putative Roles in Regulating Terpenoid Biosynthesis, *PLoS One; 10*(9): e0139002. doi: 10.1371/journal.pone.0139002.
- Liu, D., Li, S., Chen, W., Zhang, B., Liu, D., Liu, B. (2016). Transcriptome Analysis of Purple Pericarps in Common Wheat (Triticum aestivum L.). PLoS ONE 11(5): e0155428. <https://doi.org/10.1371/journal.pone.0155428>.
- Sun, L., Wang, J., Song, K., Sun, Y., Qin, Q., Xue, Y. (2019). Transcriptome analysis of rice (*Oryza sativa* L.) shoots responsive to cadmium stress, *Scientific Reports 9*;10177.
- Wu, L., Li, M., Tian, L., Wang, S., Wu, L., Ku, L. (2017) Global transcriptome analysis of the maize (*Zea mays* L.) inbred line 08LF during leaf senescence initiated by pollination-prevention. *PLoS ONE 12*(10): e0185838. https://doi.org/10.1371/journal.pone.0185838.
- Tombuloglu, G., Tombuloglu, H., Sakcali, M. S., Unver, T. (2015). High-throughput transcriptome analysis of barley (*Hordeum vulgare*) exposed to excessive boron, *Gene, 15*; 557(1):71-81. doi: 10.1016/j.gene.2014.12.012. Epub.
- Dugas, D. V., Monaco, M. K., Olsen, A., Klein, R. R., Kumari, S., Ware, D., Klein, P. E. (2011). Functional annotation of the transcriptome of *Sorghum bicolor* in response to osmotic stress and abscisic acid. *BMC Genomics 12*:514. doi: 10.1186/1471-2164-12-514.
- Dudhate, A., Shinde, H., Tsugama, D., Liu, S., Takano, T. (2018). Transcriptomic analysis reveals the differentially expressed genes and pathways involved in drought tolerance in pearl millet [*Pennisetum glaucum* (L.) R. Br]. *PLoS ONE 13*(4): e0195908. <https://doi.org/10.1371/journal.pone.0195908>.
- Parvathi, M. S., Karaba, N., Nanja, Y. A., Mahantesha, B. N., Gowda, M. V. (2019). Transcriptome analysis of finger millet (*Eleusine coracana* L.) reveals unique drought responsive genes. *Journal of Genetics, 98*(2), 46, doi:10.1007/s12041-019-1087-0.
- Zheng, X., Pan, C., Diao, Y., You, Y., Yang, C., Hu, Z. (2013). Development of microsatellite markers by transcriptome sequencing in two species of *Amorphophallus* (*Araceae*), *BMC Genomics 14*; 490.
- Tafolla-Arellano, J. C. (2017). Transcriptome Analysis of Mango (*Mangifera indica* L.) Fruit Epidermal Peel to Identify Putative Cuticle-Associated Genes. *Sci. Rep. 7*, 46163; doi: 10.1038/srep46163.
- Mittal, A., Yadav, I., Arora, N., Boora, R., Mittal, M., Kaur, P., Erskine, W., Chhuneja, P., Singh, I., Gill, M., Singh, K. (2020). RNA-sequencing based gene expression landscape of guava cv. Allahabad Safeda and comparative analysis to colored cultivars, *BMC Genomics 21*: 484.
- Kaur, K., Awasthi, P., Tiwari, S. (2021). Comparative transcriptome analysis of unripe and ripe banana (cv. *Nendran*) unraveling genes involved in ripening and other related processes. *PLoS ONE 16*(7): e0254709. <https://doi.org/10.1371/journal.pone.0254709>.
- Hill, M. G., Wurms, K. V., Davy, M. W., Gould, E., Allan, A., Mauchline, N. A. (2015). Transcriptome Analysis of Kiwifruit (*Actinidia chinensis*) Bark in Response to Armoured Scale Insect (*Hemiberlesia lataniae*) Feeding. *PLoS ONE 10*(11): e0141664. <https://doi.org/10.1371/journal.pone.0141664>.
- El-Sharkawy, I., Liang, D., Xu, K. (2015). Transcriptome analysis of an apple (*Malus×domestica*) yellow fruit somatic mutation identifies a gene network module highly associated with anthocyanin and epigenetic regulation, *Journal of Experimental Botany, 66*;(22) 7359-7376, <https://doi.org/10.1093/jxb/erv433>.
- Wang, Z., Cui, Y., Vainstein, A., Chen, S., Ma, H. (2017). Regulation of Fig (*Ficus carica* L.) Fruit Color: Metabolomic and Transcriptomic Analyses of the Flavonoid Biosynthetic Pathway, Front. Plant Sci., https://doi.org/10.3389/fpls.2017.01990.
- Vasiliki, F., Vasileios, F., Thanasis, M., Thalia, A., Irene, P., Artemios, M. B., Dimitris, K., Costas, D., Eran, P., Angelos, K. K. (2008). Transcriptome analysis approaches for the isolation of trichome-specific genes from the medicinal plant *Cistus creticus* subsp. Criticus, *Plant Mol Biol;68*(6):633-51. doi: 10.1007/s11103-008-9399-0.
- Marco, C., Paz, M., Gaetano, D., Stefano, L. M., Angela, R. P., Francisco, R. T., Manuel, T., Alessandra, G. (2012). Comparative transcriptome analysis of stylar canal cells identifies novel candidate genes implicated in the self-incompatibility response of *Citrus clementina*, *BMC Plant Biol.; 12*: 20. doi: 10.1186/1471-2229-12-20.
- Huang, G., Zeng, Y., Wei, L., Yao, Y., Dai, J., Liu, G., Gui, Z. (2020). Comparative transcriptome analysis of mulberry reveals anthocyanin biosynthesis mechanisms in black (*Morus atropurpurea* Roxb.) and white *(Morus alba* L.) fruit genotypes, *BMC Plant Biology 20*: 279.
- Qi, M., Chengjie, C., Tao, X., Aiping, L., Chaoyang, L., Yehua, H. (2018). Comprehensive tissue-specific transcriptome profiling of pineapple (*Ananas comosus*) and building an eFP-browser for further study, *PeerJ. 6*: e6028, doi: 10.7717/peerj.6028.
- Crystal, S., Darren, W., Christopher, F., Damian, D. (2012). Transcriptome analysis at four developmental stages of grape berry (*Vitis vinifera* cv. Shiraz) provides insights into regulated and coordinated gene expression, *BMC Genomics 13*: 691.
- Fang-fang, F., Ying-shu, P., Gui-bin, W. (2021). Integrative analysis of the metabolome and transcriptome reveals seed germination mechanism in *Punica granatum* L., *Journal of Integrative Agriculture, 20*(1): 132-146.
- Guo, S. (2015). Comparative transcriptome analysis of cultivated and wild watermelon during fruit development. *PloS one 10*, e0130267.
- Kaushik, P., Kumar, S. (2019). Data of *de novo* assembly of fruit transcriptome in *Aegle marmelos* L., *Data Brief.*, 104189. doi: 10.1016/j.dib.2019.104189.
- Diningrat, D. S, Widiyanto, S. M., Pancoro, A., Shim, D., Panchangam, B., Zembower, N., Carlson, J. E. (2015). Transcriptome of Teak (*Tectona grandis*, L. f) in Vegetative to Generative Stages Development, *Journal of Plant Sciences 10* (1): 1-14, ISSN 1816-4951 / DOI: 10.3923/jps.2015.1.14.
- Tin, H., So, T., Sreng, S., Thammavong, B., Boounithiphonh, C., Boshier, H. D., MacKay, J. (2020). Reference transcriptomes and comparative analyses of six species in the threatened rosewood genus Dalbergia, *Scientific Reports 10*: 17749.
- Liu, J. P., Xia, Z. Q., Tian, X. Y., Li, Y. J. (2015). Transcriptome sequencing and analysis of rubber tree (*Hevea brasiliensis* Muell.) to discover putative genes associated with tapping panel dryness (TPD), *BMC Genom.;16*:398. doi: 10.1186/s12864-015-1562-9.
- Zemao, Y., Zhigang, D., Ruike, L., Bibo, W., Qing, T., Ying, X., Chaohua, C., Jianguang, S. (2017) Transcriptome Analysis of Two Species of Jute in Response to Polyethylene Glycol (PEG)- induced Drought Stress, Scientific Reports, 7: 16565.
- Mingge, H., Xuke, L., John, Y., Xiugui, C., Xiaoge, W., Waqar, A. M., Junjuan, W., Delong, W., Shuai, W., Lixue, G., Chao, C., Ruifeng, C., Xiaoming, Y., Wuwei, Y. (2019). Transcriptome Analysis Reveals Cotton (*Gossypium hirsutum*) Genes That Are Differentially Expressed in Cadmium Stress Tolerance, *Int J Mol Sci., 20*(6): 1479. doi: 10.3390/ijms20061479.
- Alexey, A., Dmitriev, R. O., Novakovskiy, E. N., Pushkova, T. A., Rozhmina, A. A., Zhuchenko, L., Bolsheva, A. D., Beniaminov, V. A., Mitkevich, L. V., Povkhova, E. M., Dvorianinova, A. S., Anna, V. K., George, S. K., Nataliya, V. (2020). Transcriptomes of Different Tissues of Flax (*Linum usitatissimum* L.) Cultivars with Diverse Characteristics, *Front. Genet.*, https://doi.org/10.3389/fgene.2020.565146.
- Biswas, P., Chakraborty, S., Dutta, S., Pal, A., Das, M. (2016). Bamboo Flowering from the Perspective of Comparative Genomics and Transcriptomics, *Front. Plant Sci.*, https://doi.org/10.3389/fpls.2016.01900.
- Fan, H., Xiao, Y., Yang, Y., Xia, W., Mason, A. S., Xia, Z. (2013). RNA-Seq Analysis of *Cocos nucifera*: Transcriptome Sequencing and *De Novo* Assembly for Subsequent Functional Genomics Approaches. *PLoS ONE 8*(3): e59997. https://doi.org/10.1371/journal.pone.0059997.
- Muhammad, B. S., Ahmad, Z., Rashid, B., Hassan, S., Per, L. G., Maria, D. L., Nagy, I., Torben, A., Tayyab, H. (2019). *De novo* assembly of *Agave sisalana* transcriptome in response to drought stress provides insight into the tolerance mechanisms, *Scientific Reports 9*: 396.
- Braich, S., Baillie, C. R., Jewell, L. S., Spangenberg, G. C., Cogan, N. I. (2019). Generation of a Comprehensive Transcriptome Atlas and Transcriptome Dynamics in Medicinal Cannabis, *Scientific Reports 9*: 16583.
- Tian, X., Wang, Z., Zhang, Q., Ci, H., Wang, P., Yu, L. (2018). Genome-wide transcriptome analysis of the salt stress tolerance mechanism in *Rosa chinensis*. *PLoS ONE 13*(7): e0200938. <https://doi.org/10.1371/journal.pone.0200938>.
- Alice, T., Giacomo, C., Donald, A. H., Paolo, V., Antonio, F. (2016). Spatial and temporal transcriptome changes occurring during flower opening and senescence of the ephemeral hibiscus flower, *Hibiscus rosa-sinensis*, *Journal of Experimental Botany*, doi:10.1093/jxb/erw295.
- Zhao, Q., Li, R., Zhang, Y., Huang, K., Wang, W., & Li, J. (2018). Transcriptome analysis reveals in vitro-cultured regeneration bulbs as a promising source for targeted Fritillaria cirrhosa steroidal alkaloid biosynthesis. *3 Biotech*, *8*(4), 1-10.
- Ai, Y., Zhang, Q., Wang, W., Zhang, C., Cao, Z., Bao, M., & He, Y. (2016). Transcriptomic analysis of differentially expressed genes during flower organ development in genetic male sterile and male fertile *Tagetes erecta* by digital gene-expression profiling. *PLoS one*, *11*(3), e0150892.
- Yong-Hua, L., Wei, Z., Yong, L. (2015). Transcriptomic Analysis of Flower Blooming in *Jasminum sambac* through *De Novo* RNA Sequencing, *Molecules, 20*(6), 10734-10747; https://doi.org/10.3390/molecules200610734
- Li, Y., Wang, X., Chen, T., Yao, F., Li, C., Tang, Q., Sun, M., Sun, G., Hu, S., Yu, J., Song, S. (2015). RNA-Seq Based *De Novo* Transcriptome Assembly and Gene Discovery of *Cistanche deserticola* Fleshy Stem, *PLoS One.; 10*(5): e0125722. doi: 10.1371/journal.pone.0125722.
- Wang, H., Jiang, J., Chen, S., Qi, X., Peng, H., Li, P. (2013). Next-generation sequencing of the *Chrysanthemum nankingense* (*Asteraceae*) transcriptome permits large-scale unigenes assembly and SSR marker discovery. *PLoS ONE 8*:e62293. doi: 10.1371/journal.pone.0062293.
- Mei, Y., Lingping, Z., Cheng, P., Liming, X., Yanling, L., Weidong, K., Pingfang, Y. (2015). Transcriptomic Analysis of the Regulation of Rhizome Formation in Temperate and Tropical Lotus (*Nelumbo nucifera*), *Scientific Reports 5*: 13059.
- Hu, Z., Tang, B., Wu, Q., Zheng, J., Leng, P., Zhang, K. (2017). Transcriptome Sequencing Analysis Reveals a Difference in Monoterpene Biosynthesis between Scented *Lilium ‘Siberia’* and Unscented Lilium ‘Novano’, *Front. Plant Sci.*, <https://doi.org/10.3389/fpls.2017.01351>.
- Lulin, H., Xiao, Y., Pei, S., Wen, T., Shangqin, H. (2012). The first Illumina-based *de novo* transcriptome sequencing and analysis of safflower flowers. *PloS one, 7*(6):e38653. doi: 10.1371/journal.pone.0038653.
- Xiao, M., Zhang, Y., Chen, X., Lee, E. J., Barber, C. J., Chakrabarty, R., Desgagne-Penix, I., Haslam, T. M., Kim, Y. B., Liu, E. (2013). Transcriptome analysis based on next-generation sequencing of non-model plants producing specialized metabolites of biotechnological interest. *J Biotechnol 166*: 122-134.
- Shi, D., Wang, J., Bai, Y., Liu, Y. (2020). Transcriptome sequencing of okra (*Abelmoschus esculentus* L. Moench) uncovers differently expressed genes responding to drought stress, *Journal of Plant Biochemistry and Biotechnology 29*, 170.
- Han, J., Thamilarasan, S. K., Natarajan, S., Park, J. I., Chung, M. Y., Nou, I. S. (2016). *De Novo* Assembly and Transcriptome Analysis of Bulb Onion (*Allium cepa* L.) during Cold Acclimation Using Contrasting Genotypes. *PLoS ONE 11*(9): e0161987. https://doi.org/10.1371/journal.pone.0161987.
- Mayer, E. S. (2015). Effects of different temperature regimes on flower development, microsporogenesis and fertility in bolting garlic (*Allium sativum*). *Funct Plant Biol. 42*(6):514-526. doi: 10.1071/FP14262.
- Nie, S., Li, C., Xu, L., Wang, Y., Huang, D., Muleke, E. M. (2015). *De novo* transcriptome analysis and identification of critical genes involved in bolting and flowering in radish (*Raphanus sativus* L.). *BMC Genomics, 17.* doi.10.1186/s12864-016-2633-2.
- Geng, M., Sabine, K. C., Soren, K. R. (2020). Transcriptome Analysis Reveals Candidate Genes Related to Anthocyanin Biosynthesis in Different Carrot Genotypes and Tissues, *Plants, 9*(3), 344; https://doi.org/10.3390/plants9030344.
- Jie, L. (2016). Transcriptome Analysis of Pepper (*Capsicum annuum*) Revealed a Role of 24-Epibrassinolide in Response to Chilling, *Front. Plant Sci*, https://doi.org/10.3389/fpls.2016.01281.
- Yusong, J., Qinhong, L., Yong, Z., Yiqing, L., Jianbi, L. (2017). Transcriptome analysis reveals the genetic basis underlying the biosynthesis of volatile oil, gingerols, and diarylheptanoids in ginger (*Zingiber officinale* Rosc.), *Botanical Studies 58*: 41.
- Qing, L., Yuzhi, Q., Xinxi, H., Guangcun, L., Hongying, D., Xingyao, X., Wanxing, W. (2020). Transcriptome analysis uncovers the gene expression profile of salt-stressed potato (*Solanum tuberosum* L.), *Scientific Reports 10*: 5411.
- Zhan, Y., Qu, Y., Zhu, L., Shen, C., Feng, X., Yu, C. (2018). Transcriptome analysis of tomato (*Solanum lycopersicum* L.) shoots reveals a crosstalk between auxin and strigolactone. *PLoS ONE 13*(7): e0201124. https://doi.org/10.1371/journal.pone.0201124.
- Zhang, C., Zheng, H., Wu, X., Xu, H., Han, K., Peng, J., Lu, Y., Lin, L., Xu, P., Wu, X. (2018). Genome-wide identification of new reference genes for RT-qPCR normalization in CGMMV-infected *Lagenaria siceraria*. Peer J., 6, e5642.
- Sun, Y., Zhang, H., Dong, W., He, S., Qiao, S., Qi, X., Hu, Q. (2022). Integrated analysis of the transcriptome, sRNAome, and degradome reveals the network regulating fruit skin coloration in sponge gourd (*Luffa acutangula*), *Scientific Reports 12*; 3338.
- Shukla, A., Singh, V. K., Bharadwaj, D. R., Kumar, R., Rai, A. (2015). *De Novo* Assembly of Bitter Gourd Transcriptomes: Gene Expression and Sequence Variations in Gynoecious and Monoecious Lines. *PLoS ONE 10*(6): e0128331. https://doi.org/10.1371/journal.pone.0128331.
- Guo, W. L., Chen, B. H., Chen, X. J., Guo, Y. Y., Yang, H. L., Li, X. Z. (2018). Transcriptome profiling of pumpkin (*Cucurbita moschata* Duch.) leaves infected with powdery mildew. *PLoS ONE 13*(1): e0190175. https://doi.org/10.1371/journal.pone.0190175.
- Xiaolei, S., Jing, N., Xin, L., Michael, J. S., Cankui, Z., Yi, Z., Si, M., Nan, S., Zhangjun, F., Robert, T., Zhenxian, Z. (2018). Transcriptomic and functional analysis of cucumber (*Cucumis sativus* L.) fruit phloem during early development, *The Plant Journal, 96*, 5 p. 982-996, https://doi.org/10.1111/tpj.14084.
- Hu, L., Wu, G., Hao, C., Yu, H., Tan, L. (2016). Transcriptome and selected metabolite analyses reveal points of sugar metabolism in jackfruit (*Artocarpus heterophyllus* Lam.)., *Plant Science, 248*; 45-56. doi: 10.1016/j.plantsci.2016.04.009.
- Ashrafi, H., Hill, T., Stoffel, K., Kozik, A., Yao, J., Chin-Wo, S. R. (2012). *De novo* assembly of the pepper transcriptome (*Capsicum annuum*): a benchmark for *in silico* discovery of SNPs, SSRs and candidate genes. *BMC Genomics.;13*(1):571. pmid:23110314.
- Chenxi, X., Chen, J., Honghe, S., Xiaofeng, C., Xiaoli, W., Chenhui, G., Yi, Z., Wenli, L., Xuepeng, S., Yimin, X., Jie, D., Zhonghua, Z., Sanwen, H., Shaojun, D., Beiquan, M., Quanxi, W., Zhangjun, F., Quanhua, W. (2017). Draft genome of spinach and transcriptome diversity of 120 Spinacia accessions, *Nature Communications* 8; 15275.
- Vaidya, K., Ghosh, A., Kumar, V., Chaudhary, S., Srivastava, N., Tanushree, K., Tiwari, S. (2013). *De Novo* Transcriptome Sequencing in *Trigonella foenum-graecum* L. to Identify Genes Involved in the Biosynthesis of Diosgenin, *The Plant Genome, 6*, 2. <https://doi.org/10.3835/plantgenome2012.08.0021>.
- Su-Young, H., Kyeong-Sik, C., Ki-Oug, Y., Hyun-Oh, L., Kwang-Soo, C., Jong-Taek, S., Su-Jeong, K., Jeong-Hwan, N., Hwang-Bae, S., Yul-Ho, K. (2017). Complete Chloroplast Genome Sequences and Comparative Analysis of *Chenopodium quinoa* and *C. album*, *Front. Plant Sci*., https://doi.org/10.3389/fpls.2017.01696.
- Choudhary, S., Mahantesha, B. N., Sharma, N., Singh, R. D., Gopal, L. (2019). Transcriptome profiling of coriander: a dual-purpose crop unravels stem gall resistance genes, *J Genet, 98*:19.
- Gille, S. (2011). Deep sequencing of voodoo lily (*Amorphophallus konjac*): An approach to identify relevant genes involved in the synthesis of the hemicellulose glucomannan. *Planta 234*, 515-526. https://doi.org/10.1007/s00425-011-1422-z.
- Wang, X. W., Luan, J. B., Li, J. M., Bao, Y. Y., Zhang, C. X., Liu, S. S. (2010). *De novo* characterization of a whitefly transcriptome and analysis of its gene expression during development. *BMC Genomics., 11*: 400-10.1186/1471-2164-11-400.
- Mutasa-Gottgens, E. S., Joshi, A., Holmes, H. F., Hedden, P., Gottgens, B. (2012). A new RNASeq-based reference transcriptome for sugar beet and its application in transcriptome-scale analysis of vernalization and gibberellin responses. *BMC Genomics 13*:99. https://doi.org/10.1186/1471-2164-13-99.
